# Effect-Directed, Chemical and Taxonomic Profiling of Peppermint Proprietary Varieties and Corresponding Leaf Extracts †

**DOI:** 10.3390/antiox12020476

**Published:** 2023-02-14

**Authors:** Antonio M. Inarejos-Garcia, Julia Heil, Patricia Martorell, Beatriz Álvarez, Silvia Llopis, Ines Helbig, Jie Liu, Bryon Quebbeman, Tim Nemeth, Deven Holmgren, Gertrud E. Morlock

**Affiliations:** 1 Department of Functional Extracts, ADM Valencia, 46740 Carcaixent, Spain; 2 Chair of Food Science, Institute of Nutritional Science, and TransMIT Center for Effect-Directed Analysis, Justus Liebig University Giessen, Heinrich-Buff-Ring 26-32, 35392 Giessen, Germany; 3 Archer Daniels Midland, Nutrition, Health&Wellness, ADM® Biopolis S.L. Parc Cintífic Universitat de València, Calle Catedrático Agustín Escardino Benlloch, 46980 Paterna, Spain; 4 Department of Science & Technology, ADM Wild Europe, 13597 Berlin, Germany; 5 Department of Genetics, ADM Wild, Chicago, IL 60601, USA

**Keywords:** antioxidant, flavonoid, functional food, planar bioassay, planar enzyme assay, high-performance thin-layer chromatography, *Caenorhabditis elegans*, HPTLC–UV/Vis/FLD–EDA, HPLC–PDA/MS, headspace SPME–GC–FID/MS

## Abstract

During the development of novel, standardized peppermint extracts targeting functional applications, it is critical to adequately characterize raw material plant sources to assure quality and consistency of the end-product. This study aimed to characterize existing and proprietary, newly bred varieties of peppermint and their corresponding aqueous extract products. Taxonomy was confirmed through genetic authenticity assessment. Non-target effect-directed profiling was developed using high-performance thin-layer chromatography–multi-imaging–effect-directed assays (HPTLC–UV/Vis/FLD–EDA). Results demonstrated substantial differences in compounds associated with functional attributes, notably antioxidant potential, between the peppermint samples. Further chemical analysis by high-performance liquid chromatography–photodiode array/mass spectrometry detection (HPLC–PDA/MS) and headspace solid-phase microextraction–gas chromatography–flame ionization/MS detection (headspace SPME–GC–FID/MS) confirmed compositional differences. A broad variability in the contents of flavonoids and volatiles was observed. The peppermint samples were further screened for their antioxidant potential using the *Caenorhabditis elegans* model, and the results indicated concordance with observed content differences of the identified functional compounds. These results documented variability among raw materials of peppermint leaves, which can yield highly variable extract products that may result in differing effects on functional targets in vivo. Hence, product standardization via effect-directed profiles is proposed as an appropriate tool.

## 1. Introduction

Plants from *Mentha* species (Lamiaceae) are globally widespread and broadly consumed worldwide for its pleasant flavor, alone or mixed with other herbs [1]. Mentha is derived from the Greek Mintha, the name of a mythical nymph who metamorphosed into this plant [2] and its species name piperita is from the Latin piper meaning pepper, which indicates its aromatic and pungent taste [3]. *Mentha* × *piperita* L. is the natural hybrid from *M. aquatica* and *M. spicata* [4], and is widely identified as peppermint. It is a perennial herb native to Europe, naturalized in the North of America, and cultivated in many parts of the world [5].

Apart from the flavoring properties, phytotraditional uses of pepermint leaves or its essential oil (non-polar part) are related to respiratory and/or digestive diseases [6], with the essential oil being the most widely used component of peppermint. Most of the clinical human trials performed have investigated the effect of peppermint oil for therapeutic applications [5,6,7] in gastrointestinal (17 trials), respiratory tract (8 trials), analgesic (4 trials) and bioavailability/metabolism (1 trial) studies. Therefore, more than 90% of clinical human trials were performed with peppermint oil, whereby 60% of them were related to gastrointestinal actions [5]. Although main volatile components of peppermint oil (i.e., menthol, menthone, D-neomenthol, eucalyptol, menthyl acetate, and piperitone) are functional compounds [6,8], one of the main drawbacks of using its essential oil as functional ingredient is its toxicity [8], and therefore, daily dosage must be controlled. In contrast, aqueous peppermint extracts were studied in acute toxicity in mice, and there was observed no deaths at highest dosages by oral administration, and thus, the corresponding report concluded safety of aqueous peppermint extracts [9].

Peppermint leaves are licensed in Germany as a standard medicinal tea to treat dyspepsia [5], but only few peppermint extracts are commercially available and literature on standardization of its polar extracts is limited. The presence of various functional molecules in the polar fraction of peppermint leaves indicate that the corresponding extracts must also be considered as functional phytochemical products. The polar extract of peppermint shows different flavonoids such as flavones (i.e., luteolin glycosides, and isorhoifolin), flavanones (i.e., eriodictyol, and naringenin derivatives), and flavonols (i.e., quercetin, rutin and glycosides) [6]. Therefore, a good compromise between polar and non-polar bioactive components is desirable for a peppermint extract.

The current demand for peppermint is expected to grow by 7.1% per year from 2022 to 2032 [10]. For a sustainable production, the development of proprietary peppermint varieties with higher yield, disease resistance, climate resilience, and unique functional ingredients is necessary. Genotyping analysis by means of restriction-site associated DNA sequencing [11] is effective and efficient to determine the genetic identity and relationships in proprietary peppermint varieties. The large numbers of single-nucleotide polymorphism (SNP) markers generated from restriction-site associated DNA sequencing (RAD-Seq) provide a better understanding of genetic diversity and identification.

The control of peppermint raw materials together with a fine selection of peppermint leaves is mandatory to develop novel standardized peppermint extracts with adequate composition of value-adding compounds, such as bioactive compounds, flavonoids, and volatiles, targeting functional applications [12]. The main objective of this study is to analyze, compare and characterize conventional and new proprietary peppermint leaves and their corresponding aqueous extracts. Therefore, an effect-directed profiling was developed using high-performance thin-layer chromatography–multi-imaging–effect-directed assays (HPTLC–UV/Vis/FLD–EDA). Twelve different effect-directed assays were applied and the resulting effect-profiles were compared. The obtained antioxidative profiles were compared with results from the respective *Caenorhabditis elegans* method. Further chemical analysis was performed by high-performance liquid chromatography–photodiode array detection/mass spectrometry (HPLC–PDA/MS) and headspace solid-phase microextraction–gas chromatography–flame ionization/MS detection (headspace SPME–GC–FID/MS). The genetic assessment was performed to ensure the proper taxonomy.

## 2. Materials and Methods

### 2.1. Chemicals and Materials

#### 2.1.1. HPTLC–UV/Vis/FLD–EDA

Bidistilled water was produced by a Heraeus Destamat Bi–18E, Thermo Fisher Scientific, Dreieich, Germany. *n*-Hexane, toluene, ethyl acetate, ethanol, methanol, formic acid, dimethyl sulfoxide (all chromatography grade), α-glucosidase (from *Saccharomyces cerevisiae*), β-glucuronidase (from *Escherichia coli*), acetylcholinesterase (from *Electrophorus electricus*), butyrylcholinesterase (from horse serum), tyrosinase (from mushroom), Müller–Hinton broth (for microbiology), caffeine (≤100%), acarbose (for pharm.), rivastigmine tartrate (≥98%), imidazol (≥99.5%), Tween 20, lysogeny broth (Lennox) powder (including 5 g/L sodium chloride), ampicillin sodium salt, D-(+)-glucose (99.5%) and various buffer salts (specified in respective assay literature) were provided by Fluka Sigma–Aldrich, Steinheim, Germany. β-Glucosidase (from almonds) was obtained from ABCR, Karlsruhe, Germany. 5-Bromo-4-chloro-3-indolyl β-D-glucuronide sodium salt (X-glucuronide, ≥98%) was delivered from Carbosynth, Compton–Berkshire, UK. Tetracycline hydrochloride (reagent grade) was provided by Serva Electrophoresis, Heidelberg, Germany. (2S)-2-Amino-3-(3,4-dihydroxyphenyl)propanoic acid (levodopa, 99%) was delivered by J&K Chemicals Chandigarh, India. 17β-Estradiol (≥98.5%) was bought from Dr. Ehrenstorfer, Augsburg, Germany. D-Saccharolactone and fluorescein di(β-D-galactopyranoside) were purchased from Santa Cruz Biotechnology, Dallas, TX, USA. 4-Nitroquinoline 1-oxide (4-NQO, ≥98%) was obtained from TCI, Eschborn, Germany. Fast Blue B salt (95%) was bought from MP Biomedicals, Eschwege, Germany. 1-Naphthyl acetate was purchased from Panreac, Barcelona, Spain. 2,2-Diphenyl-1-picrylhydrazyl (DPPH•, 95%) was obtained by Alfa Aesar, Schwerte, Germany. Koji acid (>98%), gallic acid (≥98%), bovine serum albumin (fraction V, ≥98%), 3-(4,5-dimethylthiazolyl-2)-2,5-diphenyl-2H-tetrazolium bromide (MTT, ≥98%), 2-naphthyl-β-D-glucopyranoside, natural product reagent A (≥98%), polyethylene glycol (PEG) 8000 (Ph. Eur.), 3-[(3-cholamidopropyl) dimethylammonium]-1-propanesulfonate (CHAPS, ≥98%), tris(hydroxymethyl)aminomethane (Tris, ≥99.9%), anisaldehyde (4-methoxybenzaldehyd), sulfuric acid, glacial acetic acid (both >98%), hydrochloric acid (HCl, 37%), apigenin (ROTICHROM TLC) and various salts for buffer and media were from Carl Roth, Karlsruhe, Germany. 2-Naphthyl-α-D-glucopyranoside (99%, Fluorochem) were from VWR, Darmstadt, Germany. *Saccharomyces cerevisiae* BJ3505 equipped with the human estrogen receptor were obtained from D. P. McDonnel et al. [13,14]. Additional chemicals for the respective medium and substrate solution were described elsewhere [15]. *Saccharomyces cerevisiae* BJ1991 equipped with the human androgen receptor were delivered by Xenometrix, Allschwil, Switzerland. Additional chemicals for the respective medium and substrate solution were described elsewhere [16,17]. Degalan P 28 N was obtained by Röhm, Darmstadt, Germany. Dulbecco’s phosphate-buffered saline was from Biochrom, Berlin, Germany. *Salmonella typhimurium* TA1535/pSK1002 cryostock was purchased from Trinova Biochem, Giessen, Germany. Additional chemicals and reagents for the respective solutions were described elsewhere [18,19]. The Gram-negative, bioluminescent marine *Aliivibrio fischeri* bacteria (DSM–7151) were bought form the German Collection of Microorganisms and Cell Cultures, Berlin, Germany; chemicals for the medium were described elsewhere [20]. Gram-positive soil bacteria *Bacillus subtilis* (Bundesgesundheitsamt, BGA) spore suspension, HPTLC plates silica gel 60, and the same with acid-stable fluorescence indicator F_254_ s (for comparison, also plates with F_254_), all 20 cm × 10 cm, were provided by Merck, Darmstadt, Germany. Additional chemicals for the respective *B. subtilis* medium and substrate solution were described elsewhere [21]. Rosmarinic acid (99%), eriocitrin (96%), luteolin-7-*O*-glucoside (94%), were obtained from by PhytoLab, Vestenbergsgreuth, Germany. Industrial peppermint products, i.e., different minced dried green leaf samples (L1–L8, Table 1) and the respective water extracts as dried crystalline brown powder (E1–E8), were obtained from ADM Valencia, Carcaixent, Spain. European mint leaves were grown in Germany last 2021 (L1, L2 and L8), and USA proprietary mint leaves (L3–L7, Table 1) were grown in Oregon during the same season.

#### 2.1.2. HPLC–PDA/MS

Eriocitrin, eriodictyol-7-*O*-glucoside, luteolin-7-*O*-glucuronide, luteolin-7-*O*-rutinoside, luteolin-7-*O*-glucoside, isorhoifolin, caffeic acid, rosmarinic acid, naringenin were purchased from Merck, Phytolab, and Extrasynthese, Genay, France. Formic acid, dimethyl sulfoxide (DMSO), acetonitrile, methanol and water of chromatographic quality were purchased from VWR.

#### 2.1.3. *Caenorhabditis elegans* Strain and Maintenance

*Caenorhabditis elegans* strain N2 (*C. elegans* var. Bristol, wild-type) was obtained from the *Caenorhabditis* Genetics Center, University of Minnesota, Minneapolis, MN, USA, and maintained at 20 °C on Nematode Growth Medium (NGM) plates with *Escherichia coli* strain OP50 as normal diet for nematodes.

#### 2.1.4. Genetic Assessment

ADM *Mentha* germplasm collection, Corvallis, OR, USA, is a valuable source of diversity for genetic and functional ingredient studies and for mint breeding. As plant materials, 24 mint varieties belonging to *M. x piperita*, *M. canadensis* or *M. arvensis* were from ADM *Mentha* germplasm collection including several proprietary varieties employing the Tajima-Nei model [22] (Table S1).

### 2.2. Solutions Prepared

#### 2.2.1. HPTLC–UV/Vis/FLD–EDA

Solutions of rosmarinic acid, eriocitrin, luteolin-7-*O*-glucoside, and apigenin in methanol (1 mg/mL each), whereby luteolin-7-*O*-glucoside was first dissolved with 200 µL dimethyl sulfoxide, were ultrasonicated for 3 min. Assay buffer solutions and positive control (PC) solutions were prepared as specified.

#### 2.2.2. HPLC–PDA/MS

Three standard solutions (1 mg/mL each) of eriocitrin, rosmarinic acid and luteolin-7-*O*-glucoside in DMSO were prepared by ultrasonication for 5 min. External calibration curves were assessed with at least five different calibration points (r > 0.99) for the quantification of the main groups of components in peppermint leaves and the corresponding extracts, respectively flavanones, flavones and caffeic acid derivatives.

### 2.3. Sample Preparation

#### 2.3.1. HPTLC–UV/Vis/FLD–EDA

An aliquot of each homogenized peppermint leaf (Table 1; 1 g/10 mL) or extract (0.15 g/1.5 mL) sample was extracted at an 1:10 drug/extractant ratio or dissolved at 100 mg/mL with ethanol—ethyl acetate—water 1:1:1 (*V*/*V*/*V*). Each suspension was ultrasonicated for 30 min (Sonorex Digiplus, Bandelin, Berlin, Germany) and centrifuged either at 3000× *g* for 15 min for leaf samples (Heraeus Labofuge 400) or 17,000× *g* for 5 min for extract samples (Heraeus Pico 17 Centrifuge, both Thermo Fisher Scientific). Each supernatant was transferred to an autosampler vial and stored at −20 °C.

#### 2.3.2. HPLC–PDA/MS

The peppermint leaves and corresponding extracts (Table 1) were extracted/dissolved in DMSO (10 mg/mL) in 10-mL centrifuge tubes, followed by shaking via an orbital shaker (Grant bio PSU-10i, VWR) at maximum velocity for 30 min, and filtered (0.45 µm syringe nylon filter, VWR). For MS analysis, samples were extracted/dissolved as mentioned, but in methanol instead of DMSO.

#### 2.3.3. Headspace SPME–GC–FID/MS

Each peppermint leaf (0.5 g/5 mL) or extract (0.2 g/2 mL; Table 1) sample was extracted/dissolved in water (100 mg/mL) and placed into a 20-mL headspace vial sealed with a silicone septum. Water blanks were analyzed in the beginning of the sequence and between samples.

#### 2.3.4. *Caenorhabditis elegans* Method

Powdered peppermint extracts E1–E7 were dissolved in water and respective stock solutions were prepared. Then, serial dilutions were performed. Different doses were added on NGM medium surface to get the final doses in the plate (0.1–10 mg/mL).

#### 2.3.5. Genetic Assessment

Total genomic DNA was isolated from 200 mg fresh leaves following a modified cetyltrimethylammonium bromide (CTAB) procedure [23].

### 2.4. Methods

#### 2.4.1. HPTLC–UV/Vis/FLD–EDA

HPTLC plates silica gel 60 F_254_ s and HPTLC plates silica gel 60, the latter for the SOS-Umu-C, pYAES and pYAAS bioassays, were prewashed via development with methanol—water 4:1 (*V*/*V*; Simultan Separating Chamber, biostep, Burkhardtsdorf, Germany), dried at 120 °C for 20 min (Heating Oven, Memmert, Schwabach, Germany), covered by a clean glass plate and wrapped in aluminum foil for storage in a desiccator [24]. HPTLC instruments (all CAMAG, Muttenz, Switzerland) were operated and data processed with visionCATS version 3.0 software. Application parameters (Automatic TLC Sampler 4) were as follows: 8 mm band, track distance 11 mm, distance from lower edge 8 mm and from left edge 17.5 mm, dosage speed 200 nL/s, filling vacuum time 1 s, rinsing vacuum time 6 s, rinsing cycles 3, filling cycle 1, and return unused sample into vial.

If not stated otherwise, each peppermint sample solution (10 µL/band L1–L7, and 2 µL/band E1–E7) and the standard mixture (M; generated by overspraying 1.5 µg/band each of rosmarinic acid, eriocitrin, luteolin-7-O-glucoside, and apigenin) were applied and dried in a stream of cold air (hair dryer) for 0.5 min. For the DPPH• assay, sample volumes were reduced (1 µL/band L1–L7, 0.2 µL/band E1–E7, and 0.5 µg/band M), and for the SOS-Umu-C/pYAES/pYAAS assays, increased (15 µL/band L1–L7, 3 µL/band E1–E8, and 2.3 µg/band M). The plate was developed with 7 mL ethyl acetate—toluene—formic acid—water 8:2:1.5:1 (*V*/*V*/*V*/*V*) [20] up to a migration distance of 70 mm, measured from the lower plate edge (Twin Trough Chamber, 20 cm × 10 cm), followed by drying in a stream of cold air for 1 min (hair dryer) and then 15 min (via molecular sieve, Automated Development Chamber 2). For SOS-Umu-C/pYAES/pYAAS assay, the mobile phase was adjusted to be acid-free (ethyl acetate—toluene—methanol—water 4:1:1:0.4, *V*/*V*/*V*). The relative humidity during development was 25% ± 5%. Each chromatogram was detected (TLC Visualizer) at 254 nm (UV), 366 nm (FLD) and white light illumination (Vis).

**Figure 1:**
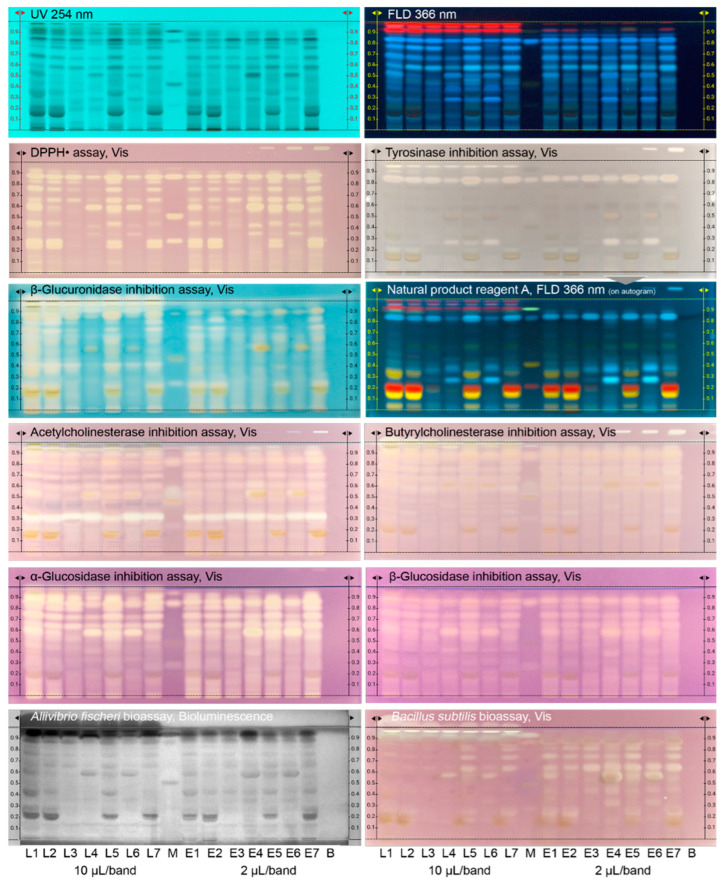
Bioprofiling of 14 peppermint products for 9 different bioactivity mechanisms: HPTLC–Vis/UV/FLD chromatograms of the various leaf samples L1–L7 (Table 1; all extracted/dissolved with water—ethanol—ethyl acetate 1:1:1; 10 µL/band each) and respective powdered extracts E1–E7 (2 µL/band each) along with standard mixture (M; eriocitrin, luteolin-7-*O*-glucoside, rosmarinic acid, and apigenin, 1.5 µg/band each) and the respective solvent blank (B) separated on HPTLC plate silica gel 60 F_254_ s with 7 mL ethyl acetate—toluene—formic acid—water 8:2:1.5:1 and detected at UV 254 nm, FLD 366 nm and after the respective (bio)assay at white light illumination (bioluminescence depicted as greyscale image for *A. fischeri*). The respective PC was applied on the upper right plate edge (cropped for some assay images). Derivatization with natural product reagent A was performed after the assay on the tyrosinase inhibition autogram.

For effect-directed assay detection, 12 analogous chromatograms were prepared with adaptations for some assays as mentioned. The respective PC for each assay was applied on the upper right plate edge (some PCs were not cut off and evident as three increasing bands in Figure 1), or for duplex assays, as agonist stipe along each separated sample track. Assay solutions/suspensions were piezoelectrically sprayed (level 5, Derivatizer) using different nozzles as specified. In case of the acidic development, the plate was neutralized by spraying (yellow nozzle) with disodium hydrogen phosphate buffer (8 g in 60 mL water, citric acid 0.1 M added to reach pH 7.5, filled up to 100 mL), followed by plate drying for 4 min (hair dryer). Note that plate neutralization was not needed for the DPPH• assay. For incubation, the plate was placed horizontally in a pre-moistened polypropylene box (27 cm × 16 cm × 10 cm, KIS, ABM, Wolframs–Eschenbach, Germany) as described [15,16,17,18,19,21]. The resulting (bio)autograms were dried (4 min, cold airstream, hairdryer) and documented (TLC Visualizer/BioLuminizer). For DPPH• assay and *B. subtilis* bioassay, the response signals increase over time and should be compared after one day.

(1) For the DPPH• assay [25,26,27], 3.0 mL 0.1% methanolic DPPH• solution with 10% 10 mM sodium chloride solution were sprayed (green nozzle). The PC was gallic acid (0.5, 1.3 and 2 µL/band, 0.1 mg/mL in methanol).
(2) For the tyrosinase inhibition assay [21], 2.0 mL levodopa substrate solution (4.5 mg/mL in phosphate buffer, 20 mM, pH 6.8, plus 2.5 mg CHAPS and 7.5 mg PEG 8000), and after drying (1 min), 2.0 mL tyrosinase solution (400 U/mL in phosphate buffer, 20 mM, pH 6.8) were sprayed (blue nozzle), followed by incubation at room temperature for 15–20 min. The PC was kojic acid (1, 3 and 6 µL/band, 0.1 mg/mL in ethanol).
(3) For the β-glucuronidase inhibition assay [28], 2.0 mL β-glucuronidase solution (50 U/mL in potassium phosphate buffer, pH 7.0), and after incubation at 37 °C for 15 min, 1.5 mL X-glucuronide solution (2 mg/mL in water) were sprayed (yellow, then red nozzle), and incubated at 37 °C for 1 h. The PC was D-saccharolactone (0.8, 1.5 and 3 µL/band, 0.1 mg/mL in water).
(4) For the acetylcholinesterase inhibition assay [21], 3.0 mL acetylcholinesterase solution (6.66 U/mL in Tris–HCl buffer plus 1 mg bovine serum albumin), followed by incubation at 37 °C for 25 min, and then 0.5 mL substrate solution (1:2 each of 3 mg/mL 1-naphthyl acetate solution in ethanol and Fast Blue B salt solution in water) were sprayed (green nozzle). The PC was rivastigmine (2, 4 and 8 µL/band, 0.1 mg/mL in methanol).
(5) For the butyrylcholinesterase inhibition assay, the same workflow was applied as in (4) but the enzyme solution was 3.34 U/mL.
(6) For the α-glucosidase inhibition assay [21], 2 mL 2-naphthyl-α-D-glucopyranoside substrate solution (12 mg in 10 mL ethanol with 10% 10 mM sodium chloride solution) and after drying (2 min), 2.5 mL α-glucosidase solution (10 U/mL in sodium acetate buffer, pH 7.5) were sprayed (yellow nozzle), followed by incubation at 37 °C for 15 min. Then, 0.75 mL chromogenic reagent Fast Blue B salt solution (2 mg/mL in water) were sprayed. The PC was acarbose (1, 3 and 6 µL/band, 3 mg/mL in methanol).
(7) For the β-glucosidase inhibition assay, the same workflow was applied as in (6) except using the 2-naphthyl-β-D-glucopyranoside substrate, β-glucosidase solution (1000 U/mL) and an incubation for 30 min. The PC was imidazole (2, 5 and 8 µL/band, 1 mg/mL in ethanol).
(8) For the *A. fischeri* bioassay [20,21], 3.5 mL bacterial suspension (150 µL bacterial cryostock incubated in 20 mL medium as specified [11] at 75 rpm and room temperature for 18–24 h) were sprayed (red nozzle). Before the green–blue bacterial luminescence was visually proven to be ready for use by shaking the culture flask in a dark room. Ten images of the still humid plate were recorded over 30 min (exposure time 60 s, trigger interval 3.0 min, BioLuminizer). The PC was caffeine (0.5, 1.5 and 3 µL/band, 1 mg/mL in methanol).
(9) For the *B. subtilis* bioassay [21], 3.5 mL bacterial suspension (100 µL bacterial cryostock in 20 mL 2.3% Müller–Hinton broth, 0.8 optical density at 600 nm) were sprayed (red nozzle), followed by incubation at 37 °C for 2 h. Then 400 µL 0.2% MTT solution in Dulbecco´s phosphate-buffered saline were sprayed (blue nozzle), followed by incubation at 37 °C for 45 min and drying at 50 °C for 5 min (TLC Plate Heater). The PC was tetracycline (0.4, 0.8 and 1.2 µL/band, 0.01 mg/mL in ethanol).
(10) For the duplex planar yeast antagonist estrogen screen (HPTLC^fix^–pYAES–FLD) bioassay [15], the agonist 17β-estradiol solution (5 µL, 0.4 ng/µL in ethanol) was applied as stripe (0.1 mm × 80 mm, width × high, FreeMode software option) along each separated sample track, followed by drying (2 min). Afterward, the plate was horizontally immersed (manually) into a Degalan solution (0.25% in *n*-hexane), dried at room temperature for 8 min and sprayed (blue nozzle) with 2.5 mL Tween 20 solution (0.05% in ethanol with 10% 10 mM sodium chloride solution). Then 2.8 mL *Saccharomyces cerevisiae* BJ3505 suspension (1 mL cryostock incubated in 29 mL medium at 100 rpm (rotatory horizontal shaker SM-30, Edmund Bühler, Bodelshausen, Germany) and 30 °C for 16 h and adjusted to 0.8 × 10^8^ cells/mL) were sprayed (red nozzle), followed by 3 h incubation at 30 °C. Then, 2 mL fluorescein di(β-D-galactopyranoside) substrate solution (5 mg in 1 mL dimethyl sulfoxide; thereof 25 µL in 2.5 mL phosphate buffer pH 7.0) was sprayed (yellow nozzle), followed by incubation at 37 °C for 0.5 h.
(11) For the duplex planar yeast antagonist androgen screen (HPTLC^fix^–pYAAS–FLD) bioassay [15], the same workflow was applied as in (10) except for *Saccharomyces cerevisiae* BJ1991 cells, testosterone solution (2 µL, 15 ng/µL in ethanol) for stripe application, 39 mL medium and 4 h incubation.
(12) For the SOS-Umu-C bioassay [16,17], the *Salmonella* TA1535/pSK1002 suspension, i.e., 25 µL cryostock in 35 mL medium (20 g/L lysogeny broth; thereof 3 mL added with 1 g D-glucose and 106 mg ampicillin; 37 °C, 16 h, 75 rpm) with 0.2 optical density at 660 nm, was sprayed (red nozzle), followed by incubation at 37 °C for 3 h and drying (4 min). Then 2.5 mL fluorescein di(β-D-galactopyranoside) substrate solution (5 mg in 1 mL dimethyl sulfoxide; thereof 25 µL in 2.5 mL phosphate buffer pH 7.0) was sprayed (yellow nozzle), followed by incubation at 37 °C for 0.5 h.

Still on the same assay plate (e.g., after tyrosinase inhibition assay and *A. fischeri* bioassay), microchemical derivatization was performed as reagent sequence using two different derivatization reagents (3 mL each, Derivatizer), i.e., natural product reagent A (1% in methanol, green nozzle), and after plate drying for 2 min and documentation, anisaldehyde sulfuric acid reagent (2% in sulfuric acid—glacial acetic acid—methanol 1:1:9, *V*/*V*/*V*, blue nozzle; followed by heating at 110 °C on plate heater for 5 min), both detected at Vis and FLD 366 nm.

#### 2.4.2. HPLC–PDA/MS

The analyses of flavanones, flavones and caffeic acid derivatives were performed according to [29]. The HPLC equipment used for the analysis consisted of a Shimadzu Nexera XR UHPLC (70 MPa) coupled to a PDA detector (SPD-M40, Izasa Scientific, Spain). Separation was performed on the octadecyl silane column Zorbax Eclipse Plus C18 (length 250 mm, ID 4.6 mm, particles 5 µm) protected by a corresponding precolumn (Agilent Technologies, Barcelona, Spain). The temperature of the column oven was 25 °C, the flow rate 1.0 mL/min, and the injection volume 2 µL. The binary gradient consisted of (A) water and (B) acetonitrile, both acidified with formic acid (0.2%, *V*/*V*), starting with A/B 90:10, increased to 25% B in 15 min, to 35% B in 17 min, and to 70% B in 19 min. For 9 min, the column was equilibrated to the initial gradient conditions. The total analysis time took 60 min. For quantification, PDA detection was performed at 280 nm using an external calibration curve with at least 5 different calibration points (r > 0.99). The results were expressed in percent (%, dry basis). The sum of flavanones (eriocitrin and eriodictyol-7-*O*-glucoside) was calculated as eriocitrin equivalents, flavones (luteolin-7-*O*-glucuronide, luteolin-7-*O*-rutinoside, luteolin-7-*O*-glucoside, and isorhoifolin) as luteolin-7-*O*-glucoside equivalents, and caffeic acid derivatives (caffeic acid and rosmarinic acid) as rosmarinic acid equivalents. For identification, the flow was set to 0.6 mL/min using a Triple TOF 5600 LC/MS/MS with software PeakView (AB SCIEX, Atlanta, GA, USA). Full mass spectra from *m*/*z* 80–1300 were recorded via electrospray ionization (ESI) in the negative ion mode using capillary voltage 3 kV, extractor voltage 4.0 V, cone voltage 30 V, ion source temperature 150 °C, desolvation temperature 300 °C, and desolvation gas flow 600 L/h.

#### 2.4.3. Headspace SPME–GC–FID/MS

Headspace SPME–GC–FID/MS analysis was performed according to [30]. By exposing the SPME fiber (2 cm; divinylbenzene/carboxen/polydimethylsiloxane 50 µm/30 µm for both latter coating; Supelco Sigma-Aldrich) to the headspace, sampling was performed at 60 °C for 1 h. Then, the adsorbed volatile components were desorbed from the fiber at 250 °C during 60 s and transferred by splitless injection (nitrogen at 1.0 mL/min) on an HP-Innowax column (ID 0.25 mm, polyethylenglycol film thickness 0.25 µm, length 60 m) installed in the GC 6890N/MSD 5973 Network (all Agilent, Waldbronn, Germany). The oven temperature ramp was as follows: 50 °C for 5 min, 3 °C/min up to 110 °C (0 min) and 5 °C/min up to 230 °C (31 min). The FID was set at 250 °C, the MSD transfer line at 280 °C, the temperature of the ionization source at 230 °C, the quadrupole at 150 °C, the electron multiplier tube to 70 eV, and the MS full scan to *m*/*z* 40–250 (acquisition rate of 10 spectral matches). Identification was performed by the NIST05 EI Database (National Institute of Standards and Technology, Gaithersburg, MD, USA). The ratio of individual components was expressed as relative area percentages. Target volatiles [6] were α-pinene, β-pinene, camphene, β-myrcene, myrcene, *cis*-carane, *p*-cymene, limonene, *cis*-β-ocymene, γ-terpinene, α-terpinene, δ-terpinene, Δ4(8) menthane, α-limonene, terpinolene, D-limonene, 1,8-cineole, *p*-cineole, *cis*-sabinene hydrate, linalool oxide, linalool, isopulegol, menthone, *iso*-menthone, menthol, terpinen-4-ol, neoisomenthol, α-terpineol, γ-terpineol, β-terpineol, *iso*-menthol, *trans*-carveol, *p*-menthone, L-menthone, menthofurane, *trans*-carveol, *cis*-carveol, pulegone, carvone, D-carvone, piperitone, *neo*-menthyl acetate, menthyl acetate, *iso*-menthyl acetate, piperitenone oxide, α-ylangene, α-copaene, β-bourbonene, β-elemene, α-caryophyllene, *trans*-β-caryophyllene, β-caryophyllene, (*Z*)-caryophyllene, β-cubebene, β-copaene, γ-muurolene, α-muurolene germacrene D, ledene, and the oxigenated sesquiterpenes spathulenol, caryophyllene oxide, piperitone oxide, and piperitenone oxide.

#### 2.4.4. *Caenorhabditis elegans* Method

For oxidative stress measurement, *Caenorhabditis elegans* strain N2 was egg-synchronized in the NGM plates (control medium) and NGM plates supplemented with different doses of peppermint extract (1, 2, 5 and 10 mg/mL, and in addition, 0.1 and 0.5 mg/mL for the peppermint extract E4) for an initial screening. Then, the optimal dose of each peppermint extract was performed by duplicate. Vitamin C (10 µg/mL) was used as an internal positive control. Nematodes survival was counted after oxidative stress (2 mM H_2_O_2_) according to [31]. Survival data were analyzed by One Way ANOVA test using a Tukey’s multiple comparison pots-test with GraphPad Prism 4 software (GraphPad Software, San Diego, CA, USA).

For *Staphylococcus aureus* infection, age-synchronized nematodes of the wild-type N2 were obtained and maintained in NGM plates, partially supplemented with a dose-response of each powdered mint extract (0.1, 0.5 and 1 mg/mL), and incubated at 20 °C. When worms reached young adult stage were transferred to the infection plates containing a lawn of *S. aureus* ATCC 25923 strain. NGM plates with the *E. coli* strain OP50 (condition without infection) and with the pathogen (infected condition) were included. Survival was scored at 25 °C during 5 days. Worms were counted as alive or dead by gentle touching with a platinum wire. Once determined the dose with the highest positive effect, a second assay was performed to confirm result at this optimal dose. Survival curves were analyzed using the log Rank T-test significance test (GraphPad Prism 4 software).

#### 2.4.5. Genetic Assessment

Both the quality and quantity of genomic DNA were evaluated by agarose gel electrophoresis and by UV/Vis spectrophotometry (NanoDrop 2000c, Thermo Fisher Scientific, Pittsburgh, PA, USA). RAD seq was performed as reported [32] with the exception that the restriction enzyme ApeKI (New England Biolabs, Ipswich, MA, USA) was used. Adapter-ligated DNA fragments were pooled and sheared to a mean size of 500 bp. The RAD-Seq libraries were enriched by polymerase chain reaction amplification and sequenced on an Illumina HiSeq 2000 (BGI, Shenzhen, China) using single-ended reads (50 bp) for each peppermint variety. For SNP calling and data analysis, the Illumina sequence reads were quality-filtered by removing the adapter sequences and reads containing greater than 50% low-quality bases. These processed reads were mapped on the reference peppermint genome using BWA-MEM (version 2, GitHub, Hobro, Denmark). Then, the comparison results filtered and SNPs were called by SAMTOOLS (version 1.16, Genome Research, Cambridgeshire, UK). High quality SNPs were used for the estimate of pairwise genetic distances and the reconstruction of phylogenetic trees based on maximum parsimony using MEGA 11 (Mega Software Technologies, Philadelphia, PA, USA) [33].

## 3. Results

### 3.1. Development of the Effect-Directed Profiling (HPTLC–UV/Vis/FLD–EDA)

Industrial peppermint products from Europe and the USA which differ regarding producer and batch (Table 1, Figure S1) were selected, i.e., 7 minced green dried leaf samples (L1–L7) and 7 brown crystalline powdered extract samples (E1–E7). The powdered extract samples were the dried water extracts industrially produced from the corresponding dried leaf samples. Both related sets of 7 samples each were compared to detect differences caused by the extraction and drying process with regard to the composition of value-adding compounds, such as bioactive compounds, flavonoids, and volatiles, important for targeting functional applications. A further powdered native extract sample E8 obtained from European peppermint leaves at industrial scale, standardized by HPLC into caffeic acid derivatives (≥1%, dry basis), flavanones (≥4%, dry basis), and flavones (≥2%, dry basis), was provided later by ADM, and in some bioassays, analyzed instead of L5 (due to limited parallel analyses per plate). The leaf samples required homogenization due to the different structural parts contained, whereas the extract samples were obtained homogenously powdered (Figure S1).

For the development of the non-target effect-directed profiling, there are no preselected target compounds in the analytical focus. The intention was a spreading of the compounds along the whole migration distance. The mobile phase system of a previous project on botanicals (ethyl acetate—toluene—formic acid—water 8:2:1.5:1) [25] was tested and found suited also for the bioprofiling of peppermint products. The separation took ca. 33 min, which was acceptable. For extraction of polyphenols, normally water or methanol were used [29]. However, another extractant, i.e., water—ethanol—ethyl acetate 1:1:1, was tested due to good experience in other projects [34]. The comparison of the different extractants for the same peppermint leaf samples L1–L7 showed that water—ethanol—ethyl acetate 1:1:1 extracted comparatively more and a broader range of polar to apolar compounds than methanol (Figure S2). The comparison of the same separation on two different plates, i.e., HPTLC plate silica gel 60 with the regular fluorescence indicator F_254_ *versus* the acid-stable F_254_ s, proved the latter to be more stable against the acidic mobile phase system and superior with regard to zone sharpness (Figure S3, especially evident at UV 254 nm and FLD 366 nm). As a starter assay, the *A. fischeri* bioassay was applied during the development of the bioprofiling (Figures S2 and S3), since it provided the most response in many other studies. Although the patterns were almost comparable, obviously the different fluorescence indicator or plate pH had an influence on the bioluminescence signal, i.e., the brilliance of the plate background was higher for the plate with F_254_. Still on the same *A. fischeri* bioassay plate (also shown after the tyrosinase inhibition assay later, Figure S6), optional derivatization was performed using two different derivatization reagents as reagent sequence, first the natural product reagent A and then anisaldehyde sulfuric acid reagent (Figures S2 and S3). The application order is explained by the increasing pH of the reagents. Including this reagent sequence, 6 different detection mechanisms were performed on the same plate, i.e., (1) Vis, (2) UV, (3) FLD, (4) effect-directed assay, (5) natural product A reagent, and (6) anisaldehyde sulfuric acid reagent.

### 3.2. Effect-Directed Profiles via HPTLC–UV/Vis/FLD–EDA

For the application of the non-target effect-directed profiling, 14 peppermint samples were applied at two different adjusted sample volumes, i.e., 10 µL/band for the leaf samples L1–L7 and 2 µL/band for the powdered extract samples E1–E7. The required lower volume was expected for the extract samples which are enriched in compounds due to the extraction/drying step and release/dissolve more compounds due to the high sample surface [34]. A respective solvent blank (B) was treated and analyzed like a sample. In the middle of the plate, a standard mixture (M) was applied with example reference compounds, such as eriocitrin, luteolin-7-*O*-glucoside, rosmarinic acid, and apigenin with increasing *hR*_F_ values (Figure S4), reported to be present as polyphenols with antioxidative activity in peppermint and related species [29]. All in all, the 16 applications filled the plate format and the samples were analyzed using the previously selected chromatographic system. The resulting chromatogram was evaluated at UV/Vis/FLD, which proved the good spread of the compounds along the migration distance (Figure 1). For the application of the 12 effect-directed assays (2 duplex bioassays included), the preparation of such a chromatogram was repeated with few adaptations for some assays as mentioned. All (bio)assay responses were repeated several times and confirmed.

After the assay application, the respective PCs showed a response in all assays, whereas the solvent blank treated like a sample did not (Figure 1). This was an important precondition for evaluation of the (bio)autograms. The applied 5-fold higher volume of the leaf samples L1–L7 had to be considered for the comparison with the powdered extract samples E1–E7. Thus, stronger intensities were given for the fine powdered crystalline extracts. The profile comparison of each minced dried leaf sample with the respective dried and powdered water extract sample showed, despite smaller differences in the response intensity, a similar profile in each pairing case (Table 1). This proved that the applied industrial leaf processing (aqueous extraction of the leaf materials) did not lose important bioactive compounds.

The DPPH• radical scavenging assay (Figure 1 and Figure S5) revealed in the 14 peppermint samples up to a dozen antioxidative compounds detected as yellow bands on a purple background. The DPPH• response was more intense after one day (Figure S5). All four reference compounds showed an antioxidative activity as expected. At the given sample amount applied, eriocitrin at *hR*_F_ 29 was visually assigned to be present (at higher amounts) in L1, L2, L5, and L7 as well as in the corresponding powdered extract samples E1, E2, E5, and E7. Rosmarinic acid at *hR*_F_ 85, though varying in the response intensity, was present in all samples. Clear differences between the samples were observed, e.g., L4 or L6 contained less and more apolar antioxidative compounds if compared to L1 or L5, which both were strongest in the overall response.

For the tyrosinase inhibition assay (Figure 1 and Figure S6), colorless (white) inhibition bands were revealed on a grey background. One prominent tyrosinase inhibitor at *hR*_F_ 85 was observed in all samples and also in the standard mixture, thus assigned as rosmarinic acid. All leaf samples L1–L7 contained another tyrosinase inhibitor at *hR*_F_ 95. Only few samples (L4–L6 and the corresponding E4–E6) showed a third tyrosinase inhibitor at *hR*_F_ 27. The given incubation at room temperature was compared with incubation at 37 °C, which was also reported as an optimal temperature for the enzyme [35], however, incubation at room temperature resulted in a superior response intensity (Figure S6). It was studied whether an autogram can be used for post-assay derivatization. The tyrosinase inhibiting plate was already 5 days old and then derivatized with the natural product A reagent detected at FLD 366 nm and white light illumination. Still sharp colorful zones were obtained (Figure 1 and Figure S6). This highlighted the potential for on-surface storage of separated samples and for multi-detection.

For the β-glucuronidase inhibition assay (Figure 1 and Figure S7), colorless (white) inhibition bands were observed on an indigo-blue colored background. Depending on the sample, up to 10 inhibition bands were revealed along the whole polarity range. All leaf samples L1–L7 contained similar to the tyrosinase inhibiting assay an additional β-glucuronidase inhibitor at *hR*_F_ 95. At the applied amounts, all four reference compounds showed β-glucuronidase inhibition, too.

For the acetylcholinesterase inhibition assay (Figure 1 and Figure S8), colorless (white) inhibition bands were detected against a purple background. One prominent acetylcholinesterase inhibitor at *hR*_F_ 32 was evident in all 14 samples. For plate neutralization, two different buffers, i.e., sodium hydrogen carbonate buffer (2.5 g in 100 mL water) *versus* disodium hydrogen phosphate buffer, were compared. The resulting autograms showed less interfering color formation for the disodium hydrogen phosphate buffer (Figure S7), which was preferred despite the comparatively three-fold higher salt load on the layer. The butyrylcholinesterase inhibition response was comparatively weaker and more in the apolar compound range (Figure 1).

For the α-glucosidase inhibition assay, colorless (white) inhibition bands on a purple background were revealed (Figure 1 and Figure S9). Up to 9 different inhibiting bands were detected, whereby the more prominent responses were observed in the middle to apolar compound range (upper autogram part). At the given amounts, three reference compounds (except for luteolin-7-*O*-glucoside) showed β-glucuronidase inhibition, too. Similar to the butyrylcholinesterase inhibition response, the β-glucosidase inhibition was comparatively weaker.

Up to 9 different antibacterial compounds acting against Gram-negative *Aliivibrio fischeri* bacteria in the samples (Figure 1 and Figure S3) were detected as dark bands (or brightened at the start zone) on the instantly bioluminescent plate background (depicted as greyscale image). However, the most prominent response was close to the solvent front. This response of at least 2 different apolar compounds was observed in all samples. All four reference compounds revealed a response at the given amount applied.

The respective sample profiles indicating antibacterial compounds acting against Gram-positive *Bacillus subtilis* bacteria were different (Figure 1), although few zones acted against both bacteria types. Up to 11 different antibacterial compounds were detected, although very different in the response intensity. Very similar to the tyrosinase inhibition autogram, one prominent sharp zone at *hR*_F_ 95 was observed in all leaf samples L1–L7. Due to the characteristic zone profile in both autograms, it was assumed to be the same, thus multi-potent, compound. All four reference compounds revealed a response at the given amount applied, whereby apigenin was strongest and clearly present in all leaf samples L1–L7.

**Figure 2:**
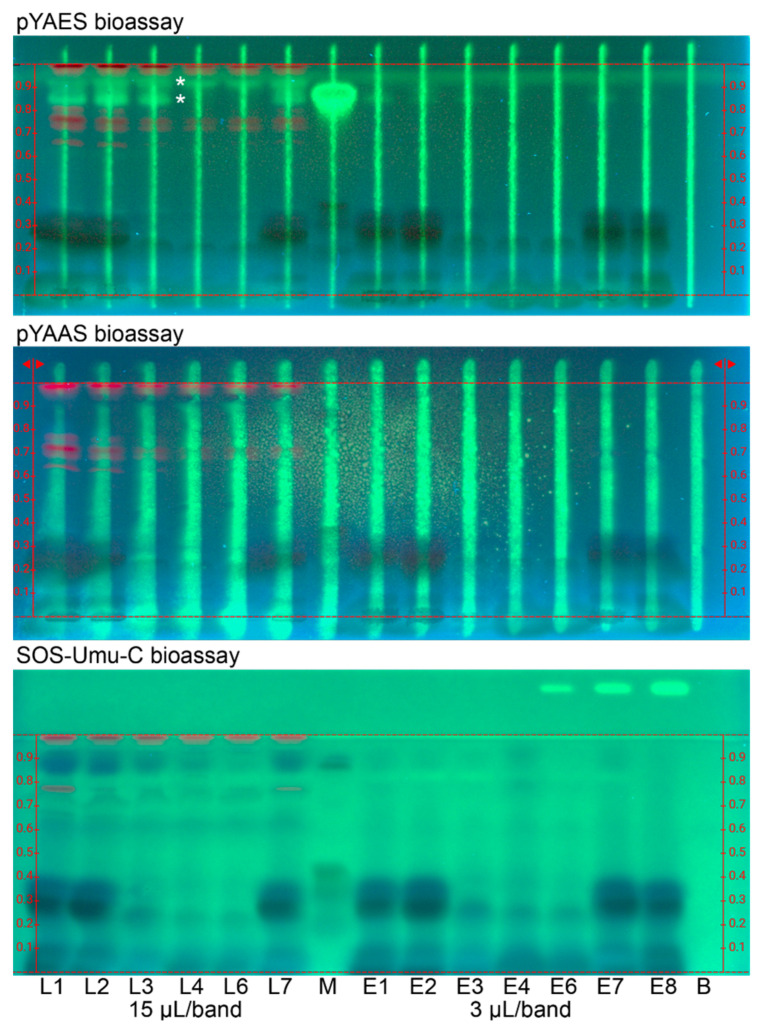
Bioprofiling for endocrine active and genotoxic compounds: HPTLC–FLD bioautograms of peppermint products (Table 1) extracted with water—ethanol—ethyl acetate 1:1:1 (0.1 mg/mL each; 15 µL/band or 1.5 mg for L1–L7, and 3 µL/band or 0.3 mg for E1–E8; L5/E5 skipped) on HPTLC plate silica gel 60 along with standard mixture (M; eriocitrin, luteolin-7-*O*-glucoside, rosmarinic acid, and apigenin, 2.3 µg/band each) and the respective solvent blank (B) separated with 7 mL ethyl acetate—toluene—methanol—water 4:1:1:0.4 and detected at 254 nm via the duplex pYAES and pYAAS bioassays as well as the planar SOS-Umu-C bioassay. Green fluorescent estrogenic compounds (*) were detected. The green fluorescence of the respective agonist stripe (applied along each sample track) or of the genotoxin 4-nitroquinoline 1-oxide (applied on the upper right plate edge) were considered as positive controls.

For the SOS-Umu-C, pYAES and pYAAS bioassays (Figure 2 and Figures S10–S12), HPTLC plates without the fluorescence indicator were used to avoid measurement signal interference since the fluorescein, which is the enzyme–substrate reaction end-product, was also detected at 254 nm. Since for all three bioassays, the same substrate was used, i.e., fluorescein di(β-D-galactopyranoside), the same green fluorescence (due to the formed fluorescein) was revealed for any agonist. Note that substrates can differ in costs by a factor of 10, and there is room for costs reduction since the substrate used here is very expensive. The mobile phase was adjusted to be acid-free (ethyl acetate—toluene—methanol –water 4:1:1:0.4) to skip the neutralization step and thus additional buffer load on the layer since the duplex bioassays required additional zone fixation. The late-delivered sample E8 (Table 1) was analyzed instead of E5. The sample volumes were increased to a maximal sample load (15 µL/band for L1–L7, 3 µL/band for E1–E8, and 2.3 µg/band each for M) to detect even traces of any estrogen, antiestrogen, androgen, antiandrogen and genotoxin. The respective green fluorescent agonist stripe was considered as the PC for the duplex assays. Antagonistic effects were observed via the signal reduction of the respective green fluorescent agonist stripe, which was applied along each sample track before the bioassay application. The detection of agonistic and antagonistic effects in the same analysis made it a duplex bioassay. However, the observation of antagonists needs further verification (V) via the pYAVES [15] and pYAVAS [17] bioassays through an additionally applied end-product stripe, which can differentiate true *versus* false-positive antagonistic effects. 

Bioprofiling for estrogenic and anti-estrogenic compounds via the duplex pYAES bioassay (Figure 2 and Figure S10) revealed up to two apolar green fluorescent estrogenic compounds in the samples, whereby the lower one was assigned to rosmarinic acid. The strong response for the given rosmarinic acid amount in the standard mixture and the missing acid in the mobile phase explained its tailing. Some anti-estrogenic compound zones were observed as signal reduction of the green fluorescent estrogen stripe. However, these antagonistic responses were not so strong and seemed to be caused by reaction with the buffer (Figure S3) since the same horizontal pattern was evident in all assays, in which the color reaction interfered (not the case for the DPPH• assay and the *Aliivibrio fischeri* bioassay). Therefore, the confirmatory pYAVES bioassay was not performed as it was not deemed necessary to do so.

Bioprofiling for androgenic and antiandrogenic compounds (Figure 2 and Figure S11) via the duplex pYAAS bioassay studied the presence of any androgens and anti-androgens. However, there were no androgens detected as green fluorescent band despite the high sample load. The proper bioassay functioning was verified by the green fluorescent testosterone stripe (applied along each sample track and considered as the PC). As already discussed for the pYAES bioassay, the same horizontal anti-androgenic compound zone pattern was observed as signal reduction of the green fluorescent testosterone stripe, which underlines our hypothesis. Also here, the respective pYAVAS bioassay was not performed.

Bioprofiling for genotoxic compounds (Figure 2 and Figure S12) via the planar SOS-Umu-C bioassay investigated the presence of any genotoxins in the industrial peppermint products. There was no green fluorescent genotoxin band revealed in the samples despite the high sample load of the dried leaf (1.5 mg for L1–L7) and powdered extract (0.3 mg for E1–E7) samples. The proper bioassay functioning was verified by the green fluorescence of the genotoxin 4-nitroquinoline 1-oxide, applied as the PC on the upper right plate edge.

### 3.3. Results of HPLC–PDA/MS Analysis

**Figure 3:**
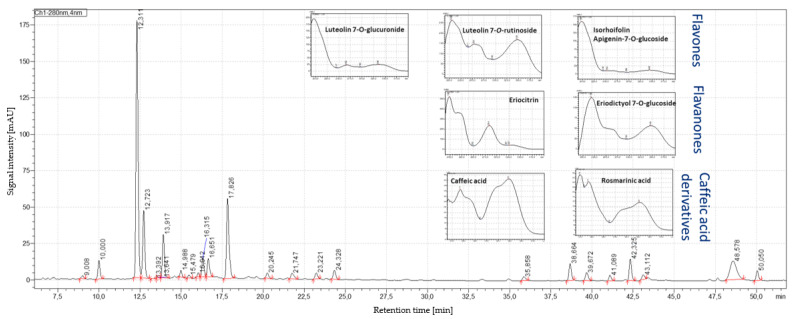
HPLC–PDA profile at 280 nm exemplarily for the peppermint leaf sample L2 from Europe, showing caffeic acid (9.01 min), eriocitrin (12.31 min), luteolin-7-*O*-rutinoside (12.72 min), eriodictyol-7-*O*-glucoside (13.39 min), luteolin-7-*O*-glucuronide (13.64 min), luteolin-7-*O*-glucoside (13.92 min), isorhoifolin (14.98 min), and rosmarinic acid (17.83 min).

**Figure 4:**
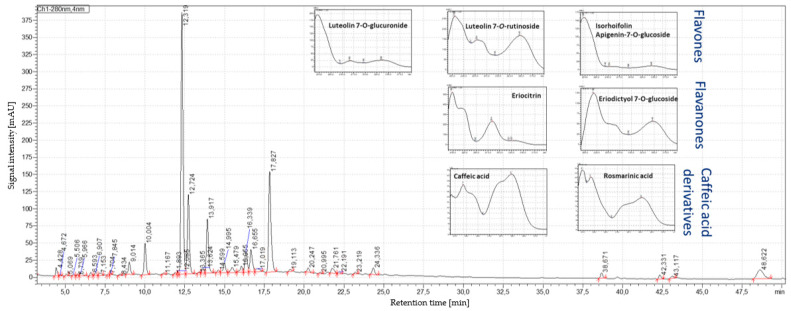
HPLC–PDA profile at 280 nm of the corresponding peppermint extract E2 from Europe.

Identification of flavanones, flavones and caffeic acid derivatives were performed by comparing retention time, PDA and MS/MS spectra with the corresponding reference standards (Table 2). A typical HPLC–PDA chromatogram at 280 nm is exemplarily illustrated for peppermint leaf sample L2 from Europe (Figure 3) and the corresponding extract E2 (Figure 4). Depending on the phenolic components, such as flavones, flavanones or caffeic acid and its derivatives, the recorded UV-Vis spectra differed.

The HPLC–QTOF-MS/MS recording in the negative ionization mode allowed the identification of the major phenolic compounds in the peppermint samples (Table 2). The flavanones eriocitrin and eriodictyol-7-*O*-glucoside eluted at 19.84 and 21.67 min, respectively. Eriocitrin showed the deprotonated molecule [M−H]^−^ at *m*/*z* 595.1665, and in the MS/MS mode, fragment ions at *m*/*z* 150.9669 and *m*/*z* 287.0000. Eriodictyol-7-*O*-glucoside showed the deprotonated molecule at *m*/*z* 449.1085 and fragments at *m*/*z* 135.0124 and *m*/*z* 150.9654. The flavones luteolin-7-*O*-rutinoside, luteolin-7-*O*-glucuronide and isorhoifolin were detected at 20.02, 21.92 and 22.33 min, respectively. Luteolin-7-*O*-rutinoside showed the deprotonated molecule at *m*/*z* 609.1459 and fragment ions at *m*/*z* 270.9649 and *m*/*z* 299.9681. Luteolin-7-*O*-glucuronide showed the deprotonated molecule at *m*/*z* 461.0721 and a fragment ion at *m*/*z* 284.9835. Isorhoifolin at *m*/*z* 577.1567 showed the fragment at *m*/*z* 268.9917. Regarding caffeic acid and its derivatives, rosmarinic acid (retention time 17.51 min) showed the deprotonated molecule at *m*/*z* 359.1775 and fragments at *m*/*z* 160.9828, *m*/*z* 132.9933 and *m*/*z* 123.0144. The deprotonated molecule of caffeic acid (retention time 27.81 min) was detected at *m*/*z* 179.0355 as base peak.

The individual composition of the major bioactive components of selected peppermint leaf samples and their corresponding extracts (all %dry basis) was determined by HPLC–PDA (Table 3). The composition of the peppermint leaf samples from USA highly fluctuated within different proprietary varieties. The content of eriocitrin ranged between 0.06% and 1.16%, whereas the European peppermint leaf samples were almost similar (1.30% to 1.43%). The highest flavanone contents (Figure S13) were observed for USA peppermint L7 (1.20%) and European peppermint L8 (1.64%). Rosmarinic acid in the peppermint leaf samples from USA (0.22% to 0.75%) also varied more than from Europe (0.27% to 0.49%). The highest flavone content (Figure S13) was observed for USA peppermint L4 (4.23%) and European peppermint L2 (1.48%). Isohoifolin and luteolin-7-*O*-glucoside showed a higher content in the USA varieties than European peppermint leaves.

For the corresponding extracts obtained from the USA and European peppermint leaves, the contents of bioactive components were significantly increased, i.e., for eriocitrin, luteolin-7-*O*-glucoside, luteolin-7-*O*-glucuronide and isohoifolin about two-fold, for rosmarinic acid more than three-fold and for eriodictyol-7-*O*-glucoside more than four-fold (Table 3). The highest flavanone contents (Figure S14) were observed for USA peppermint extract E7 (3.98%) and European peppermint extract E2 (6.79%). Also regarding the flavones, the highest contents were obtained for USA peppermint extract E7 (4.35%) and European peppermint extract E2 (6.84%).

### 3.4. Results of Headspace SPME–GC–FID/MS Analysis

The headspace SPME–GC–FID/MS analysis (Table S2) allowed for the identification of characteristic volatile components from different peppermint proprietary varieties and evaluation via their relative response proportion. A typical chromatogram of the proprietary peppermint leaf sample L5 from USA is illustrated (Figure S15). For the analyzed peppermint leaf samples (Table S2), the major monoterpenes were menthol (36.0–77.1%), followed by menthone (14.6–38.9%), neomenthol (1.6–6.0%), menthyl acetate (0.3–5.6%), and limonene (0.2–2.8%). From the sesquiterpenes, β-caryophyllene showed the highest relative response (0.1–5.4%).

Exemplarily from the European peppermint samples, the leaf sample L2 and the corresponding extract E2 were analyzed by headspace SPME–GC–FID/MS (Figures S16–S18). A similar volatile profile composition was determined for the European peppermint sample L2, if compared to the USA proprietary peppermint leaf sample L5. Major volatile component proportions were observed for menthol (41.5 %), menthone (30.7 %), neomenthol (4.1 %), menthyl acetate (5.5 %), limonene (1.5 %), and β-caryophyllene (0.2 %). Also the corresponding extract E2 showed a complex volatile composition with major characteristic peppermint volatiles found, i.e., in decreasing proportions menthyl acetate (11.8 %), menthol (6.7 %), limonene (5.6 %), β-caryophyllene (3.1 %), and menthone (1.3 %).

### 3.5. Antioxidant Activity in Caenorhabditis elegans Assay

**Figure 5:**
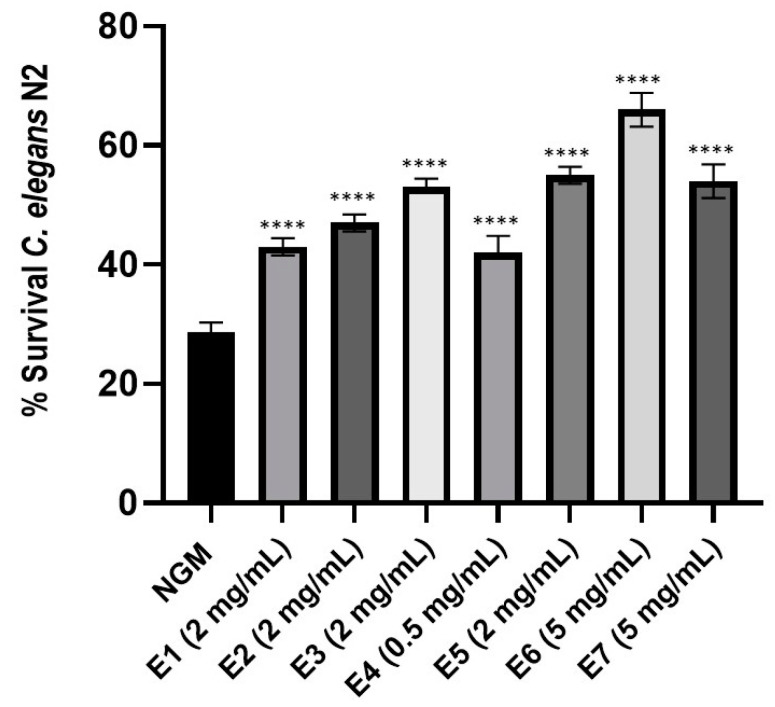
Percentage of *Caenorhabditis elegans* N2 fed with control condition (OP50) or with each peppermint extract E1–E7 at the respective optimal dose. Data correspond to the average of two independent assays. A one-way ANOVA test with a Tukey’s multiple comparison pots-test was applied. **** Significant at *p*-value < 0.0001 in comparison to NGM condition.

Previous work has been demonstrated *C. elegans* as a good model to assess antioxidant properties of oils from three *Mentha* species [36]. To determine this capacity of the peppermint extract samples E1–E7, a dose-response of each sample was performed to establish the dose with the best antioxidant activity (Figure S19). The optimal dose for each peppermint extract is indicated (Figure 5). When nematodes were fed with the different peppermint extracts and subject to acute oxidative stress, worms were more resistant to oxidative stress than control-fed nematodes (*p*-value < 0.0001). Moreover, worms treated with peppermint extract E6 showed the highest survival increase in comparison to control condition NGM (37%), while those fed with peppermint extracts E1 and E4 exhibited the lowest increase (14% and 13%, respectively).

### 3.6. Enhanced Resistance of Caenorhabditis elegans to Staphylococcus aureus Pathogen Infection

**Figure 6:**
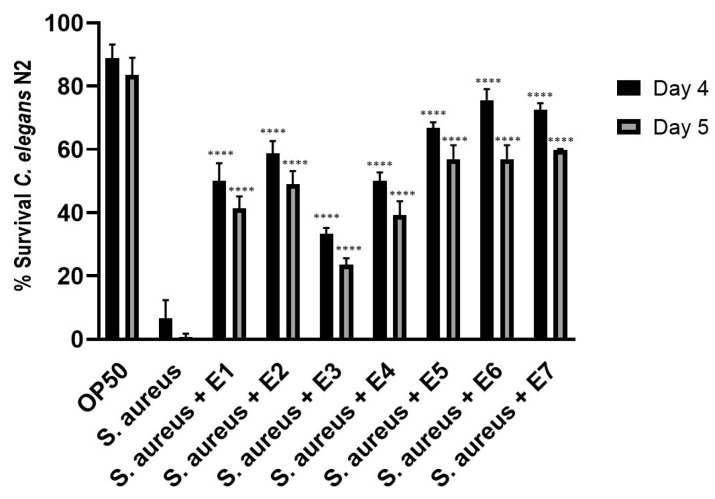
Percentage of survival of *Caenorhabditis elegans* N2 treated with the different peppermint extracts E1–E7 and infected with the pathogen *Staphylococcus aureus* (ATCC 25923) at day 4 and 5 post-infection. **** Significant *p*-value < 0.0001 in comparison to *S. aureus* condition at every corresponding day post-infection. A two-way ANOVA test was applied. Data are the average of two independent experiments.

It is known that essential oils from *Mentha* species have antimicrobial and antiviral activities [5]. A dose-response study of each peppermint extract E1–E7 was carried out in *C. elegans* on pathogen infection to determine the protection of each sample (Figure S20). All peppermint extracts increased significantly worm’s survival during pathogen challenge (*p*-value < 0.0001) in comparison to infected control condition at day 4 and 5 post-infection (Figure 6 and Figure S21). Peppermint extracts E5–E7 provided the highest pathogen resistance, followed by E2, E1 and E4. The peppermint extract E3 showed the lowest protection.

### 3.7. Genetic Assessment

The sequencing produced over 36,000,000 reads with an average of 2.5 million reads for each variety. After quality assessment and adapter trimming approximately 33,500,000 reads were used for mapping. An initial pool of 38,543 SNPs was first identified. After a quality filtering process, 15,000 SNPs were used for the data analysis.

The estimates of pairwise genetic distances among the peppermint varieties based on Tajima-Nei model [22] were obtained (Table S1). Overall, genetic distances among different varieties of *M. x piperita* were between 0.10–0.17. The peppermint leaf samples L5, L7, and MP15CS28 are genetically similar but unique to *M. x piperita* Black Mitcham. The genetic distances among different varieties of *M. canadensis* were between 0.12–0.17. L4 was a *M. canadensis* species but different from other varieties. The genetic distances between the two species were above 0.21. The genetic distances of the peppermint leaf sample L6 to all mint varieties were under 0.21 with one exception to MP981 at 0.24.

## 4. Discussion

The peppermint samples investigated (Table 1) for 14 different effects using 12 different non-target planar effect-directed assays, whereof the pYEAS and pYAAS were duplex bioassays, differed very much in their functional properties (Figure 1 and Figure 2). Hence, the developed non-target effect-directed profiling was found very informative with regard to the quality control of functional peppermint samples. Product standardization via effect-directed profiles is proposed as an appropriate tool to control all functional properties and not only targeted marker compounds, as is the case with the currently prevailing quality control.

The identification of the bioactive components in the peppermint samples by HPLC– ESI-QTOF-MS/MS revealed substances in accordance with the literature [29,37]. As expected, major flavonoids found in peppermint samples (leaves and extracts) were flavanones (eriocitrin and eriodictyol-7-*O*-glucoside), flavones (luteolin-7-*O*-rutinoside, luteolin-7-*O*-glucoside, and isorhoifolin), and hydroxycinnamic acids, in particular rosmarinic acid and caffeic acid [5,6].

The quantification of bioactive components by HPLC–PDA analysis showed a broad range of flavonoid contents, which confirms the fluctuations in value-added compounds. European and proprietary peppermint varieties from USA presented similar chromatographic profiles obtained by HPLC–PDA (Figure 3). Main differences were due to the individual content of the bioactive compounds (Table 3), which apart from the variety, are affected by different factors such as pedoclimatic and agricultural conditions [38]. The respective water extracts obtained from the different peppermint leaf samples from Europe and USA showed a similar chromatographic profile to the corresponding leaf sample (Figure 3 and Figure 4). The major bioactive components (Table 2) were extracted by the greenest solvent which is water [6]. Therefore, a preselection of compliant peppermint leaves is key to standardize the extracts into objective and primary quality markers such as flavanones, flavones and hydroxycinnamic acids as main bioactive components.

Different analytical techniques such as NMR, LC–MS, GC–MS, etc. have been employed to study water-soluble polyphenols from peppermint [6,29,39]. Nevertheless, to the best of our knowledge, the current research work covers a complete analytical study of identification and quantification by different liquid and gas chromatography techniques, together with functionality evaluation, on-surface of the separation layer and in vivo (*C. elegans*) of several *Mentha piperita* leaves and extracts, with different quality and origin.

Traditionally, the aroma composition of peppermint is identified or related with the essential oil [4,5,6], but the volatile profile is also useful to authenticate powdered extracts [30]. Therefore, the volatile profile was also analyzed in the peppermint leaf samples from USA and Europe and the corresponding powdered extracts, and showed characteristic volatile components [6,40]. Thus, additional valuable information was obtained to avoid fraud and adulteration (Figures S15–S17). Especially, the presence of characteristic volatile components in the peppermint extracts add value to the products. Several functional effects have been associated to those characteristic peppermint volatiles [5,6].

Main bioactive components in peppermint leaves are flavonoids, phenolic acids, lignan and stilbenes, and volatile components [6,29]. However, considering water-soluble (polar) polyphenols, major bioactive compounds identified in the peppermint samples were flavonoids and phenolic acids. The flavonoids were grouped into flavones (luteolin-7-*O*-rutinoside, luteolin-7-*O*-glucuronide, luteolin-7-*O*-glucoside, and isohoifolin), flavanones (eriodictyol-7-*O*-glucoside, and eriodictyol), which together with rosmarinic acid, were the major active phytochemical constituents in the leaves and the corresponding extracts. For industrial mint extracts, such as the E8, it is fundamental to evaluate the quality, and to exclude a possible fraud and adulteration. Hence, working on mint leaves (no spent mint), together with the non-drastic conditions employed during the aqueous extraction process at laboratory and industrial scales, allowed standardization of the *Mentha piperita* extracts into primary quality markers, and their comprehensive characterization by bioactivity profiles and volatiles’ fingerprints.

Phylogeny reconstruction using the 15,000 SNPs revealed genetic relationships and relatedness among the studied 24 mint varieties (Figure S22). Most mint varieties formed a distinct and well supported clade at 100%. These varieties included the peppermint leaf samples L5 and L7. Most *M. canadensis* varieties formed 2 or 3 distinct but less supported clades at below 67%. It indicated large genetic variations among these varieties. The peppermint leaf sample L6 formed a clade with mint varieties but only supported at 61%, which indicated that it might be a hybrid or cybrid between peppermint and *M. canadensis*.

The use of molecular markers for genotyping is an effective tool in peppermint breeding and variety identification and protection [41]. In this study, RAD-Seq technology was used for a rapid and cost-effective discovery of genomic SNPs from 24 mint varieties including several proprietary varieties. These SNPs have not only provided more molecular markers for peppermint genetic identification but also provided comprehensive data about the genetic diversity, divergency, and relatedness at a genome-wide scale. The RAD-Seq technology has been used for other applications ranging from QTL mapping, genome-wide association studies, and marker-assisted selective breeding. The genomic SNPs related to functional genes, especially those involved in secondary metabolic processes, can be identified and experimentally validated that will support future research on the molecular mechanism of specific traits of peppermint varieties for commercial bioactive productions.

The antioxidant capacity was in vitro evaluated by spectrophotometry via the DPPH• assay, which is linearly correlated with other antioxidant capacity indexes such as ABTS, Total Polyphenols, FRAP, and Raci [42,43]. One of the major drawbacks of in vitro assays, widely applied to evaluate antioxidant capacity, is that the oxidizing compounds used for the analyses are not present in the living being [44]. In the current research work, also in vivo studies were performed with the *C. elegans* model, which is closer to the real conditions since samples were metabolized by the living being, and the results were monitored throughout their entire life. The *C. elegans* animal model has already been used to evaluate the antioxidant properties and pathogen resistance activity of botanical extracts, among them essential oils from *Mentha* species [37,45,46]. The peppermint extracts from USA and Europe evaluated in this study enhanced worm’s survival after acute oxidative stress and *S. aureus* infection. These results are in accordance with the antioxidative and antibacterial compounds detected in the peppermint extracts in the HPTLC–UV/Vis/FLD–EDA bioprofiling. It is well known that peppermint species have benefits as natural antioxidants [36,47] and have antimicrobial properties against Gram-positive bacteria, such as *S. aureus* [48]. These properties are mainly due to phenolic compounds, such as flavonoids, and volatile compounds [49,50].

## 5. Conclusions

For quality control and standardization of plant-based products, the use of orthogonal and thus complementary analytical tools and methods (HPLC–PDA/MS, headspace SPME–GC–FID/MS, HPTLC–UV/Vis/FLD–EDA and *C. elegans* model) was crucial to cope with the sample complexity and to obtain the full picture on the 16 peppermint leaf/extract samples. Substantial differences in compounds associated with functional attributes such as flavonoids and volatiles were revealed between the peppermint samples. Especially, the side-by-side bioactivity profiles in form of an image were worth a thousand words. Non-target effect-directed analysis provided a deep understanding on the bio-functionalities of such multicomponent mixtures. Using it for industrial quality control, the costs and time per sample analysis are affordable (Euro 0.5–1.2 and 5–20 min) but vary depending on the (bio)assay, with costs for HPTLC plate and enzyme substrate being the highest expenses. The DPPH• assay and *A. fischeri* bioassay were fastest and cheapest, whereas the duplex bioassays took longest and were most expensive. Due to the step-based instrumentation, the next plate was already started when the application of the first plate was finished. Hence, for a plate handling shifted by 30 min, about 200 samples (on 12 plates) can be screened per day. New initiatives devoted to the challenges of sample complexity (www.vielstoffgemische.de) provide a good platform for exchange of knowledge.

## Figures and Tables

**Table 1:** Different peppermint products from Europe and the USA studied, i.e., the minced dried leaf samples L1–L8 and the respective powdered extract samples E1–E8 (Figure S1).

ID	Declaration of Peppermint Products
L1	EU Mint leaves 130004707
L2	EU Mint leaves 1502100065
L3	USA Mint leaves CS28
L4	USA Mint leaves MA6
L5	USA Mint leaves MP2
L6	USA Mint leaves MP11
L7	USA Mint leaves MP13
L8	EU Mint leaves ME-22/0054
E1	Extract from EU Mint leaves 130004707
E2	Extract from EU Mint leaves 1502100065
E3	Extract from USA Mint leaves CS28
E4	Extract from USA Mint leaves MA6
E5	Extract from USA Mint leaves MP2
E6	Extract from USA Mint leaves MP11
E7	Extract from USA Mint leaves MP13
E8	Extract ME-22/0054 Ref35219000560000

**Table 2:** Phenolic compounds identified in the European peppermint leaf sample L2 by HPLC–ESI-QTOF-MS/MS and HPLC–PDA.

Phenolic Compound	HPLC–ESI-QTOF-MS/MS							HPLC–PDA	
	Score	Formula	Intensity	Expected *m*/*z*	Found at *m*/*z*	Fragment Ion *m*/*z*	RT (min)	RT (min)	UV Max (nm)
Caffeic acid	90%	C_9_H_8_O_4_	2844	179.0350	1.79.0355	-	17.51	9.01	218. 323
Eriocitrin	90%	C_27_H_32_O_15_	577,510	595.1668	595.1665	150.9669, 287.0000	19.84	12.336	216. 283
Luteolin-7-*O*-rutinoside	91%	C_27_H_30_O_16_	9361	609.1461	609.1459	270.9649, 299.9681	20.02	12.747	216. 348
Eriodictyol-7-*O*-glucoside	93%	C_21_H_22_O_11_	8672	449.1089	449.1085	135.0124, 150.9654	21.67	13.42	217. 283
Luteolin-7-*O*-glucuronide	93%	C_21_H_18_O_12_	99,000	461.0725	461.0721	284.9838	21.92	13.64	255. 348
Isorhoifolin	90%	C_27_H_30_O_14_	220,895	577.1563	577.1567	268.9917	22.33	15.014	266. 336
Rosmarinic acid	90%	C_18_H_16_O_8_	21,842	359.0772	359.0775	123.0144, 132.9933, 160.9828	27.81	17.845	218. 328

**Table 3:** HPLC–PDA analysis of the major bioactive components (%, dry basis) of peppermint proprietary leaf samples L1–L8 from USA and Europe, and corresponding extracts E1–E8.

Components (%, Dry Basis)	USA					Europe		
in Leaves	L3	L4	L5	L6	L7	L8	L2	L1
Eriocitrin	0.06 ± 0.00	0.00 ± 0.00	0.62 ± 0.12	0.00 ± 0.00	1.16 ± 0.03	1.43 ± 0.05	1.40 ± 0.00	1.30 ± 0.42
Luteolin-7-*O*-rutinoside	0.16 ± 0.00	0.04 ± 0.00	0.65 ± 0.14	0.05 ± 0.01	0.78 ± 0.03	0.47 ± 0.01	0.82 ± 0.00	0.70 ± 0.32
Eriodictyol-7-*O*-glucoside	0.01 ± 0.00	0.02 ± 0.00	0.13 ± 0.03	0.01 ± 0.01	0.04 ± 0.01	0.21 ± 0.00	0.07 ± 0.00	0.10 ± 0.03
Luteolin-7-*O*-glucuronide	0.11 ± 0.00	0.02 ± 0.00	0.05 ± 0.01	0.02 ± 0.00	0.22 ± 0.00	0.05 ± 0.00	0.26 ± 0.00	0.18 ± 0.16
Luteolin-7-*O*-glucoside	1.04 ± 0.01	2.88 ± 0.12	0.12 ± 0.01	2.06 ± 0.14	0.38 ± 0.01	0.05 ± 0.01	0.09 ± 0.00	0.11 ± 0.00
Isohoifolin	1.81 ± 0.01	1.27 ± 0.07	0.18 ± 0.03	0.52 ± 0.00	1.19 ± 0.06	0.13 ± 0.01	0.24 ± 0.00	0.30 ± 0.02
Rosmarinic acid	0.75 ± 0.01	0.22 ± 0.00	0.60 ± 0.13	0.30 ± 0.05	0.75 ± 0.05	0.49 ± 0.02	0.27 ± 0.01	0.35 ± 0.05
in Extracts	E3	E4	E5	E6	E7	E8	E2	E1
Eriocitrin	0.14 ± 0.01	0.02 ± 0.01	2.05 ± 0.00	0.01 ± 0.00	3.78 ± 0.01	3.81 ± 0.04	6.38 ± 1.80	4.04 ± 0.63
Luteolin-7-*O*-rutinoside	0.30 ± 0.02	0.05 ± 0.01	2.29 ± 0.04	0.02 ± 0.00	2.39 ± 0.01	1.51 ± 0.01	3.85 ± 1.11	2.09 ± 0.34
Eriodictyol-7-*O*-glucoside	0.04 ± 0.00	0.02 ± 0.01	0.57 ± 0.00	0.00 ± 0.00	0.21 ± 0.03	0.78 ± 0.01	0.41 ± 0.13	0.75 ± 0.12
Luteolin-7-*O*-glucuronide	0.10 ± 0.00	0.01 ± 0.00	0.26 ± 0.00	0.01 ± 0.00	0.61 ± 0.03	0.24 ± 0.00	1.26 ± 0.39	0.36 ± 0.07
Luteolin-7-*O*-glucoside	0.05 ± 0.00	0.03 ± 0.01	0.34 ± 0.00	0.01 ± 0.00	0.24 ± 0.02	0.11 ± 0.03	0.27 ± 0.08	0.25 ± 0.04
Isohoifolin	1.46 ± 0.02	0.19 ± 0.05	0.54 ± 0.00	0.04 ± 0.00	0.90 ± 0.00	0.31 ± 0.00	1.05 ± 0.29	0.74 ± 0.12
Rosmarinic acid	2.32 ± 0.00	0.45 ± 0.12	2.46 ± 0.03	0.37 ± 0.00	2.57 ± 0.01	1.71 ± 0.01	1.40 ± 0.43	1.68 ± 0.27

## Data Availability

The data presented in this study are available on request from the corresponding author.

## Supplementary Materials

The following supporting information can be downloaded at: https://www.mdpi.com/article/10.3390/antiox12020476/s1, Table S1: Estimate pairwise genetic distances among peppermint varieties; Table S2: Relative proportion of volatiles of USA peppermint proprietary leaves; Figure S1: Investigated peppermint samples; Figure S2: Extractant selection for HPTLC; Figure S3: HPTLC plate selection; Figure S4: Standard mixture assignment; Figure S5: DPPH• assay; Figure S6: Tyrosinase inhibition assay; Figure S7: β-Glucuronidase inhibition assay; Figure S8: Acetylcholinesterase inhibition assay; Figure S9: α-Glucosidase inhibition assay; Figure S10: Bioprofiling for estrogenic and anti-estrogenic compounds; Figure S11: Planar bioprofiling for androgenic and antiandrogenic compounds; Figure S12: Planar bioprofiling for genotoxic compounds; Figure S13: Total content of flavones and flavanones (%, dry basis) of different peppermint leaf samples; Figure S14: Total content of flavones and flavanones (%, dry basis) of corresponding peppermint extracts; Figure S15: Headspace SPME–GC–FID profile of the corresponding proprietary peppermint leaf sample L5; Figure S16: Headspace SPME–GC–FID profile of the European peppermint leaf sample L2; Figure S17: Headspace SPME–GC–FID profile of the peppermint extract E2; Figure S18: Major volatile components of peppermint leaf sample L2 and the corresponding extract E2; Figure S19: Dose-response of the peppermint extract samples E1−E7 on pathogen protection in the *C. elegans* model; Figure S20: Dose-response of the peppermint extract samples E1−E7 on pathogen protection in the *C. elegans* model; Figure S21: Percentage of survival of *C. elegans* N2 treated with the different peppermint extracts E1−E7; Figure S22: Phylogenetic relationships of the 24 peppermint varieties inferred using the maximum parsimony.
