# Supplementary material for: Effect-Directed, Chemical and Taxonomic Profiling of Peppermint Proprietary Varieties and Corresponding Leaf Extracts
Source: Antioxidants (Basel). 2023 Feb 14;12(2):476. doi: 10.3390/antiox12020476 (PMC9952098; doi:10.3390/antiox12020476)
Supplement: Supplementary file 1 [file antioxidants-12-00476-s001.zip › antioxidants-2197594-supplementary.pdf]

Supplementary Material

Effect-directed, chemical and taxonomic profiling of peppermint proprietary varieties and corresponding leaf extracts

Antonio M. Inarejos-Garcia [1,#], Julia Heil [2], Patricia Martorell [3], Beatriz Álvarez [3], Silvia Llopis [3], Ines Helbig [4], Jie Liu [5], Bryon Quebbeman [5], Tim Nemeth [5], Deven Holmgren [5], and Gertrud Morlock [2,*,#]

Dedicated to the 85th birthday of Dr. Fred Rabel, ChromHELP, Woodbury, NJ, USA

[1] Department of Functional Extracts, ADM Valencia, 46740 Carcaixent, Spain

[2] Justus Liebig University Giessen, Institute of Nutritional Science, Chair of Food Science, and TransMIT Center for Effect-Directed Analysis, Heinrich-Buff-Ring 26-32, 35392 Giessen, Germany

[3] Physiology Department, Faculty of Medicine, Universidad Autónoma de Madrid, 28029 Madrid, Spain R&D Department Biopolis, 46980 Paterna, Spain

[4] Department of Science & Technology, ADM Wild Europe, 13597 Berlin, Germany

[5] Department of Genetics, ADM Wild, Chicago, IL, United States

* Correspondence: gertrud.morlock@uni-giessen.de

[#] Contributed equally.

This pdf contains

Tables S1 to S2

Figures S1 to S22

List of Contents

No.	Legend	Page
Table S1	Estimate pairwise genetic distances among mint varieties	S3
Table S2	Headspace SPME−GC−FID relative response proportion of volatiles of USA peppermint proprietary leaves	S4
Figure S1	Investigated peppermint samples	S5
Figure S2	Extractant selection for HPTLC profiling	S6/7
Figure S3	HPTLC plate selection	S8/9
Figure S4	Standard mixture assignment for HPTLC	S10
Figure S5	HPTLC−DPPH• assay	S11
Figure S6	HPTLC−tyrosinase inhibition assay	S12/13
Figure S7	HPTLC−β-glucuronidase inhibition assay	S14
Figure S8	HPTLC−acetylcholinesterase inhibition assay	S15
Figure S9	HPTLC−α-glucosidase inhibition assay	S16
Figure S10	Planar bioprofiling for estrogenic and anti-estrogenic compounds	S17
Figure S11	Planar bioprofiling for androgenic and antiandrogenic compounds	S18
Figure S12	Planar bioprofiling for genotoxic compounds	S19
Figure S13	HPLC−PDA spider-web diagram of total content of flavones and flavanones of peppermint leaf samples **L1**−**L8**	S20
Figure S14	HPLC−PDA spider-web diagram of total content of flavones and flavanones of peppermint extract samples **E1**−**E8**	S21
Figure S15	Headspace SPME−GC−FID profile of the proprietary peppermint leaf sample **L5**	S22
Figure S16	Headspace SPME−GC−FID profile of the European peppermint leaf sample **L2**	S22
Figure S17	Headspace SPME−GC−FID profile of the European peppermint extract sample **E2**	S23
Figure S18	Major volatile components of European peppermint leaf sample **L2** and the corresponding extract **E2**	S23
Figure S19	Dose-response of the peppermint extract samples **E1**−**E7** on pathogen protection in the *C. elegans* model	S24
Figure S20	Dose-response of the peppermint extract samples **E1**−**E7** on pathogen protection in the *C. elegans* model	S25
Figure S21	Percentage of survival of *C. elegans* N2 treated with the different peppermint extracts **E1**−**E7**	S26
Figure S22	Phylogenetic relationships of the 24 mint varieties inferred using the maximum parsimony.	S27

Table S1. Estimates of pairwise genetic distances among 24 mint varieties (among these, some of the studied ones, *i.e.* **L4–L7**, marked red) with a total of 15,000 SNPs

BlackMitcham	
MBMB	0.14
MB14AH17	0.100.11
L5	0.140.170.11
MP2B1	0.160.120.120.13
MP201B	0.140.130.100.080.09
MP1301A	0.160.140.140.160.150.13
MP1301	0.150.160.130.140.150.120.10
MP14R11	0.140.140.140.140.170.160.110.10
MP15RZ32	0.170.150.130.150.130.130.100.110.12
L7	0.140.150.140.170.140.170.140.140.150.13
MP8	0.170.180.130.170.140.140.170.170.170.170.17
MP9	0.150.160.130.120.140.120.120.120.120.110.160.15
MA13CS29	0.250.200.240.230.220.240.250.220.200.240.210.200.25
MG13SF65	0.230.200.210.170.190.170.180.200.200.200.200.170.210.14
MP15CS28	0.140.150.140.150.160.170.130.140.110.140.130.190.130.210.19
L6	0.210.190.170.180.160.150.180.150.200.180.180.150.180.160.110.20
MAID	0.220.190.190.200.180.180.170.200.200.210.180.160.170.140.100.190.12
MA1	0.240.200.210.180.180.170.210.200.200.190.230.200.180.130.100.210.110.12
L4	0.220.200.190.180.200.160.180.190.190.180.220.160.170.150.120.210.120.130.11
MA819	0.220.180.180.190.210.190.200.210.200.210.210.190.210.130.090.170.110.120.100.11
MP981	0.220.190.220.260.220.240.230.240.210.250.210.230.240.240.280.240.240.250.280.240.25
MAORG	0.230.200.180.150.190.160.210.190.180.210.220.190.180.140.100.190.090.120.080.110.090.27
MA062	0.240.200.190.230.200.180.240.210.250.220.180.180.220.120.160.220.140.120.150.130.130.210.14

Table S2. Headspace SPME−GC−FID relative response proportion of the volatile composition (%) of peppermint proprietary leaves **L5**−**L7** compared to **MP00**, all from USA

**Volatile components**	**Relative content of volatiles (%)**				**Average ± SD**	**Percentiles (%) by GC-FID**				
	**MP00**	**L5**	**L6**	**L7**		**P0**	**P25**	**P50**	**P75**	**P100**
Limonene	0.2	0.2	1.2	2.8	1.1±1.2	0.2	0.2	0.7	1.6	2.8
Eucalyptol	3.3	2.3	0.1	3.0	2.2±1.5	0.1	1.7	2.7	3.1	3.3
Terpinene	0.2	0.4	0.0	0.1	0.2±0.2	0.1	0.1	0.2	0.3	0.4
Menthone	38.9	32.6	14.6	16.1	25.6±12.0	14.6	15.8	24.4	34.2	38.9
Benzaldehyde	0.2	0.0	0.0	0.0	0.1±0.1	0.0	0.1	0.1	0.2	0.2
Menthyl acetate	5.5	5.6	0.3	0.0	3.8±3.0	0.3	2.9	5.5	5.6	5.6
β-Caryophyllene	0.4	0.1	0.0	5.4	2.2±3.0	0.1	0.2	0.4	2.9	5.4
Isopulegone	0.3	0.0	1.0	0.0	0.6±0.5	0.3	0.5	0.6	0.8	1.0
D-Neomenthol	6.0	5.0	1.6	1.6	3.5±2.3	1.6	1.6	3.3	5.2	6.0
Terpinen-1-ol-4	0.7	0.4	0.0	0.0	0.6±0.2	0.4	0.5	0.6	0.6	0.7
Menthol	36.0	47.8	77.1	47.3	52.1±17.6	36.0	44.5	47.6	55.1	77.1
p-Menth-1-en-3-one	1.2	0.7	0.3	2.0	1.1±0.7	0.3	0.6	1.0	1.4	2.0

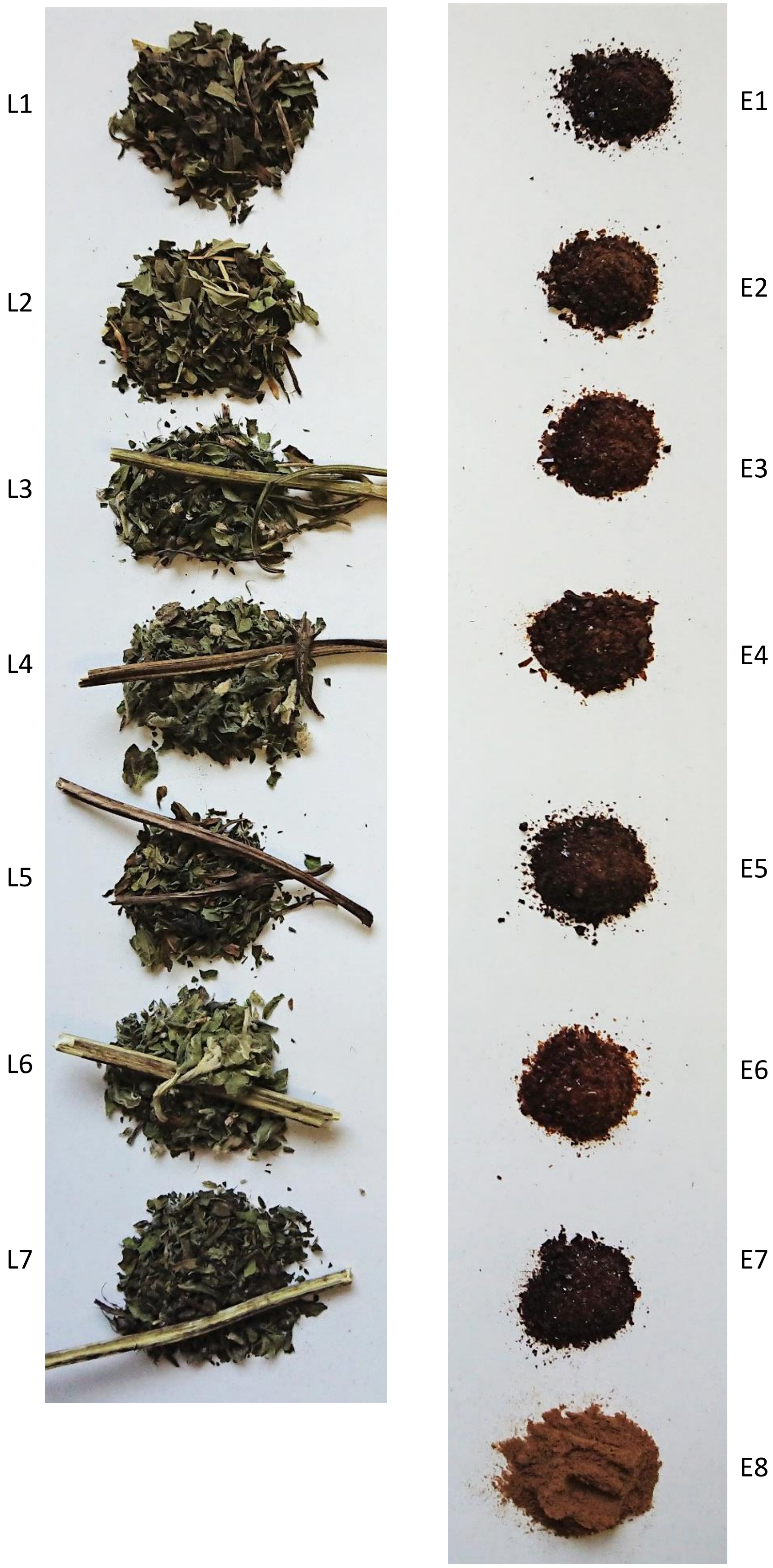

Figure S1 Investigated peppermint samples: images of the 7 minced green dried leaf samples **L1**–**L7** and 8 brown crystalline powder samples **E1**–**E8**

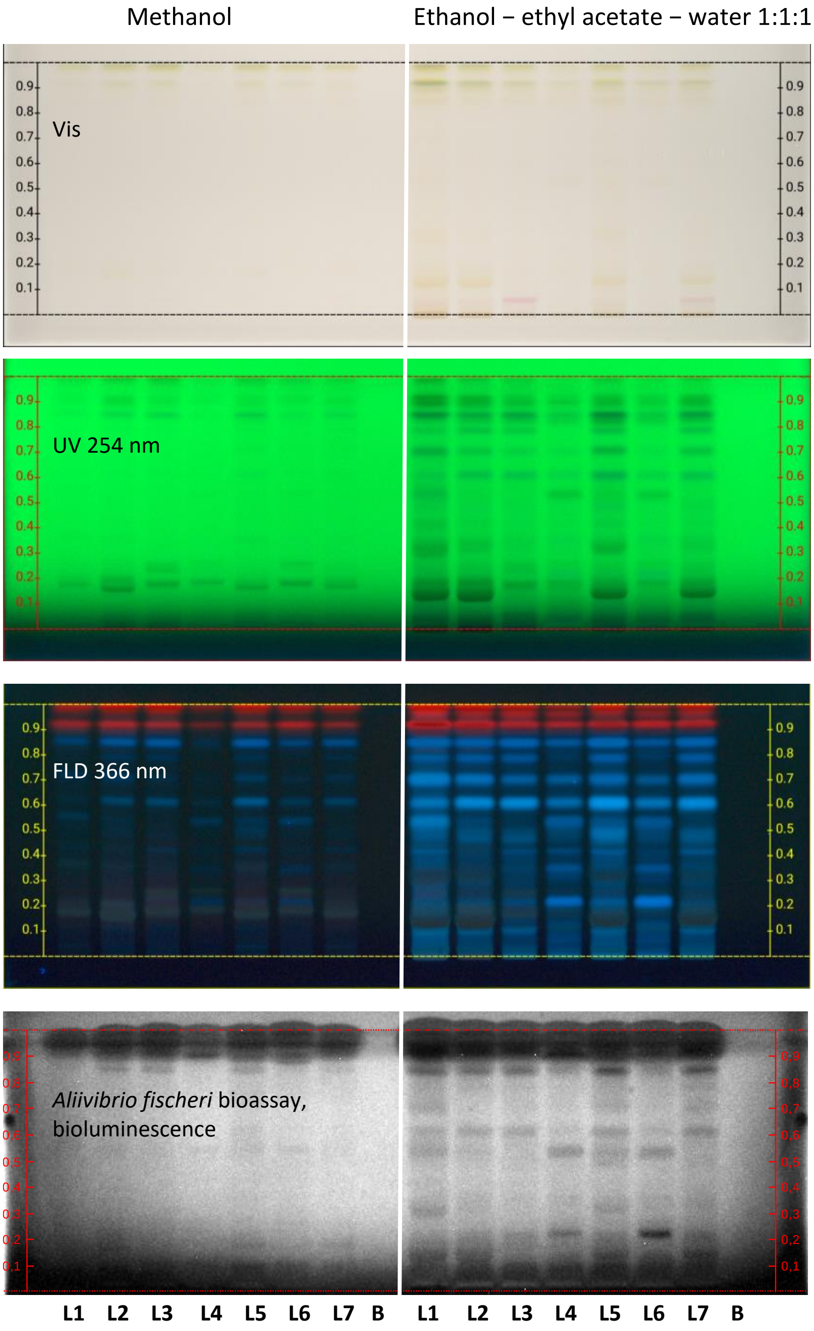

Figure S2 Extractant selection for HPTLC profiling: HPTLC–Vis/UV/FLD chromatograms showing the comparison of two different extractants for the same peppermint leaf samples **L1**–**L7** (Table 1) extracted with methanol *versus* ethanol – ethyl acetate – water 1:1:1 (0.1 mg/mL each, 10 µL/band), separated along with respective solvent blank (**B**) on HPTLC plate silica gel 60 F_{254} with 7 mL ethyl acetate – toluene – formic acid – water 8:2:1.5:1 [13] and detected at white light illumination, UV 254 nm FLD 366 nm, followed by detection of the bacterial luminescence (depicted as greyscale image) after the Gram-negative *Aliivibrio fischeri* bioassay and at white light illumination after derivatization via the natural product A reagent and then anisaldehyde sulfuric acid reagent (all detections on the same plate)

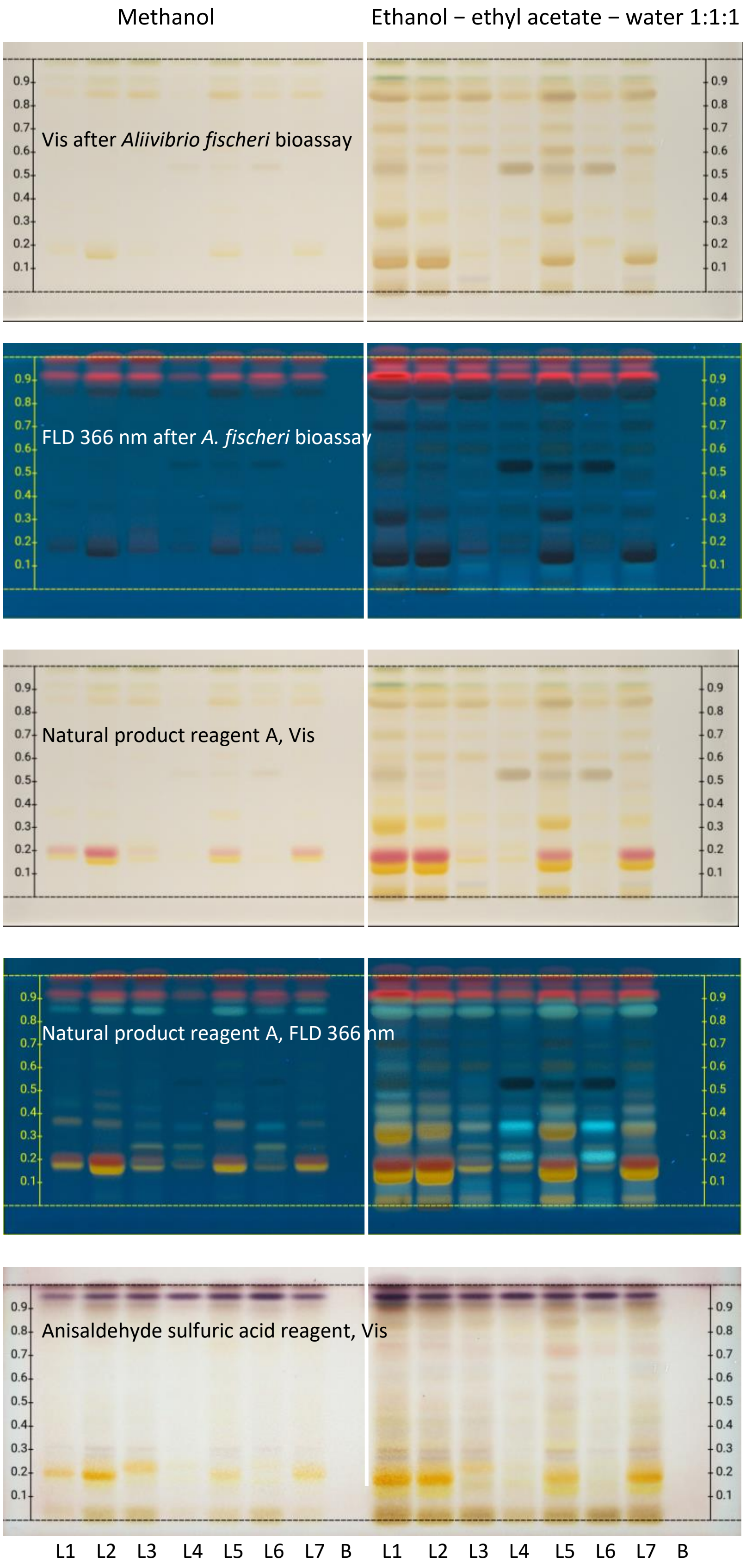

Figure S2 continued

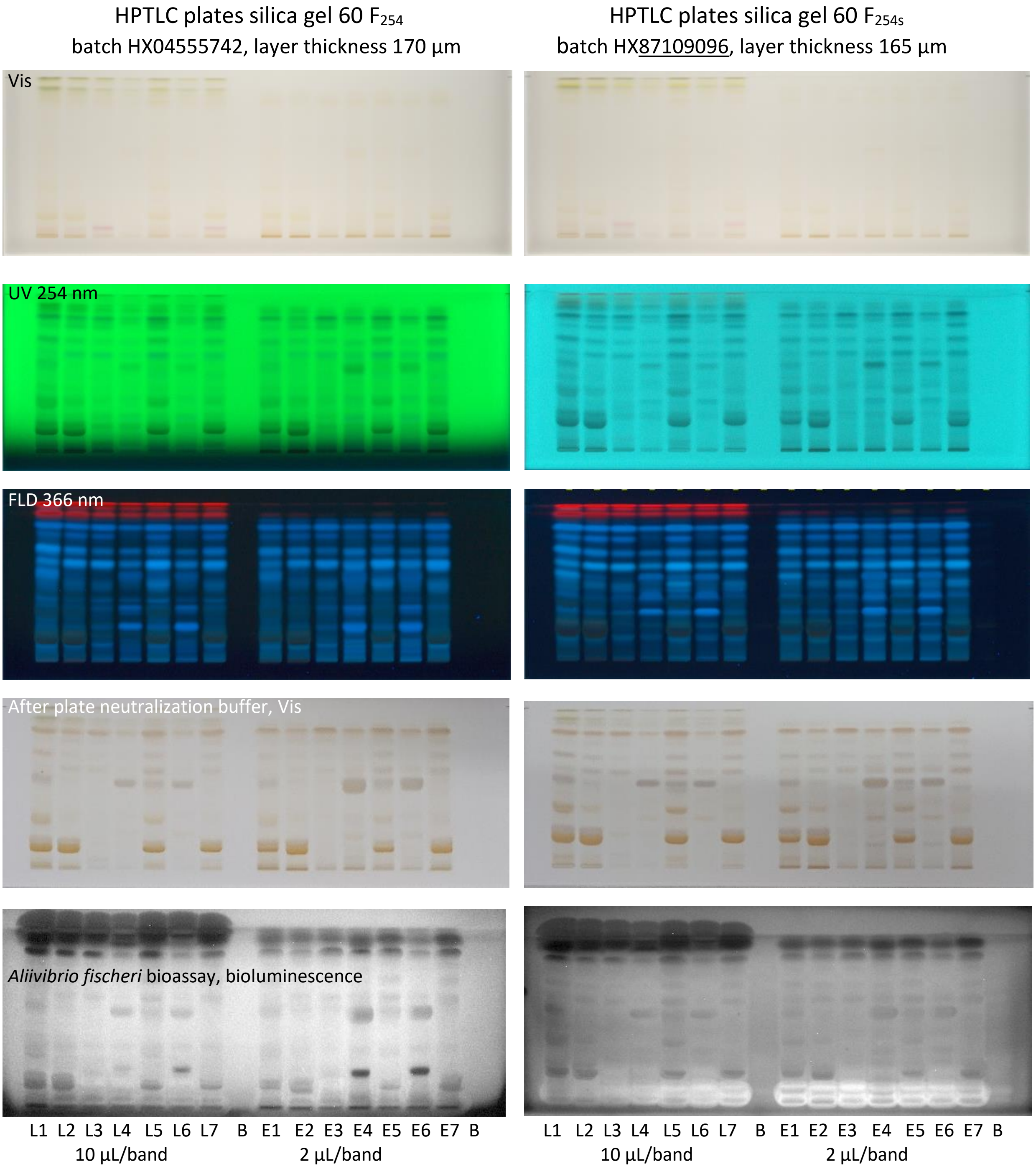

Figure S3 HPTLC plate selection: HPTLC–Vis/UV/FLD chromatograms showing the comparison of the same separation but on two different plates, *i.e.* HPTLC plate silica gel 60 with fluorescence indicator F_{254} *versus* acid-stable F_{254s}: 14 peppermint products (Table 1) extracted with ethanol – ethyl acetate – water 1:1:1 (0.1 mg/mL each; 10 µL/band for **L1**–**L7** and 2 µL/band for **E1**–**E7**) along with respective solvent blank (**B**) separated with 7 mL ethyl acetate – toluene – formic acid – water 8:2:1.5:1 and detected at white light illumination, UV 254 nm and FLD 366 nm, followed by the Gram-negative *Aliivibrio fischeri* bioassay (bacterial luminescence depicted as greyscale image), derivatization via the natural product A reagent and then anisaldehyde sulfuric acid reagent (all detections on the same plate)

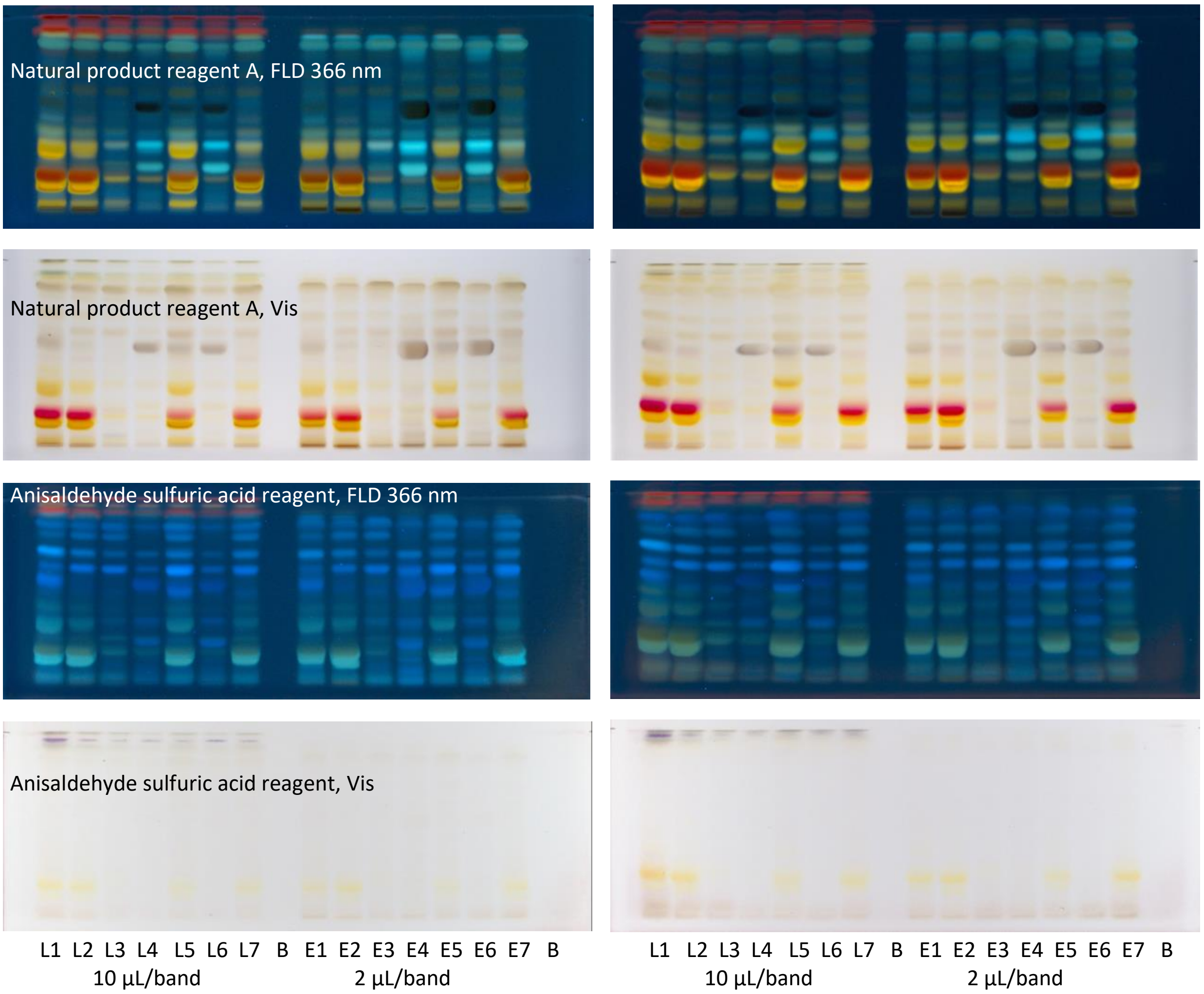

Figure S3 continued

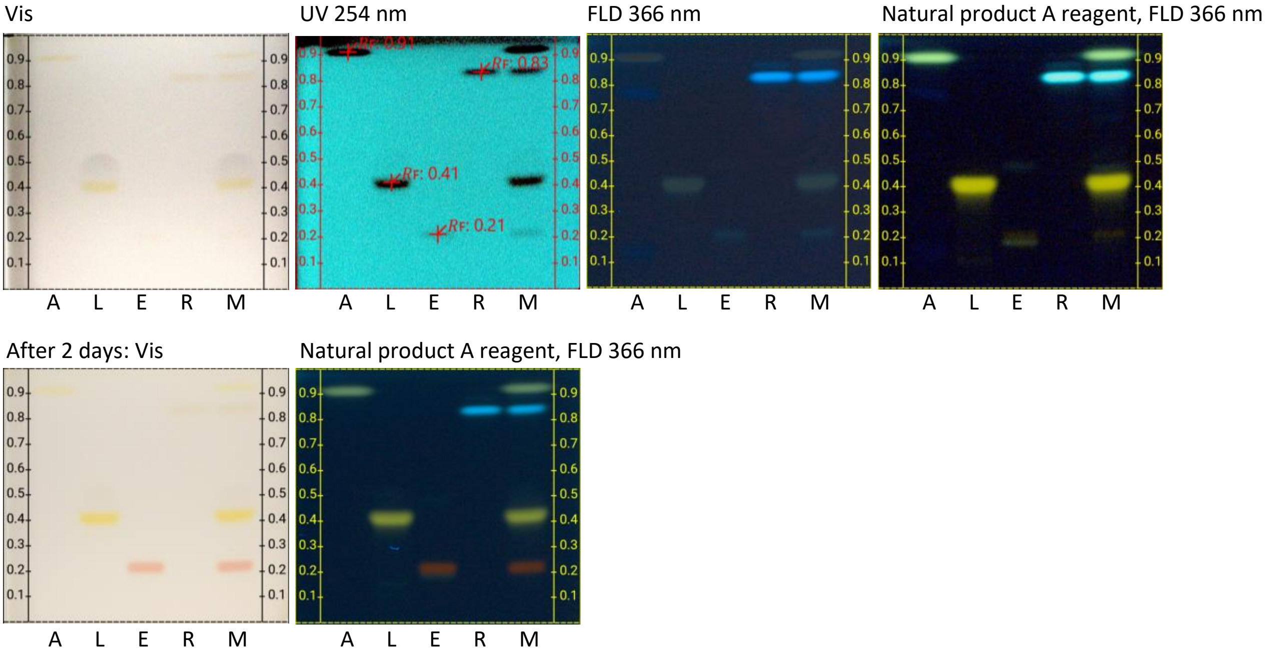

Figure S4 Standard mixture assignment: HPTLC–Vis/UV/FLD chromatograms of the individual reference compounds (1 µg/band each of apigenin **A**, luteolin-7-*O*-glucoside **L**, eriocitrin **E**, and rosmarinic acid **R**) and the oversprayed standard mixture (**M**), chromatographic system as in Figure. S3. After 2 days, eriocitrin was comparatively better detectable as rusty zone.

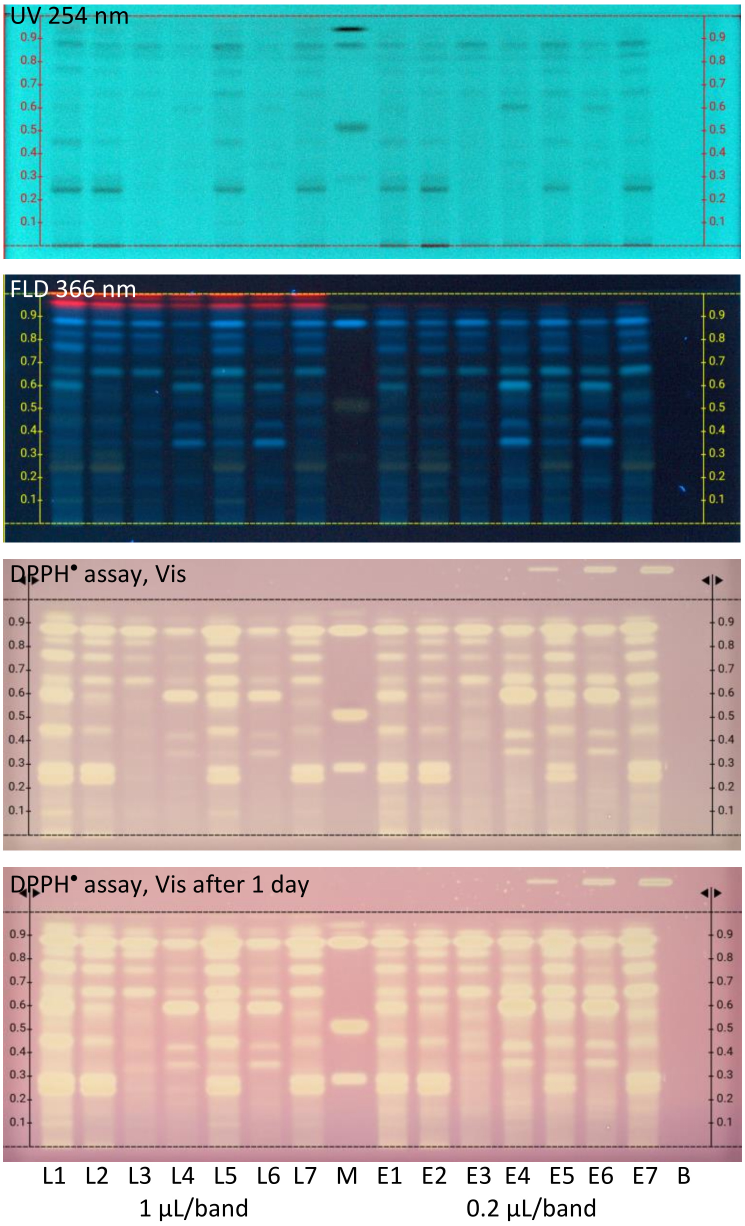

Figure S5 HPTLC–DPPH• assay: HPTLC–Vis/UV/FLD chromatograms of 14 peppermint products (Table 1) extracted with ethanol – ethyl acetate – water 1:1:1 (0.1 mg/mL each; 1 µL/band for **L1**–**L7** and 0.2 µL/band for **E1**–**E7**) on HPTLC plate silica gel 60 F_{254s} along with standard mixture (**M**; eriocitrin, luteolin-7-*O*-glucoside, rosmarinic acid, and apigenin, 0.5 µg/band each) and the respective solvent blank (**B**) separated with 7 mL ethyl acetate – toluene – formic acid – water 8:2:1.5:1 and detected at white light illumination, UV 254 nm and FLD 366 nm as well as after the DPPH• assay, detected at white light illumination. The DPPH• response was more intense after one day. The PC gallic acid was applied at 0.5, 1.3 and 2 µL/band (0.1 mg/mL in methanol) in the upper right plate.

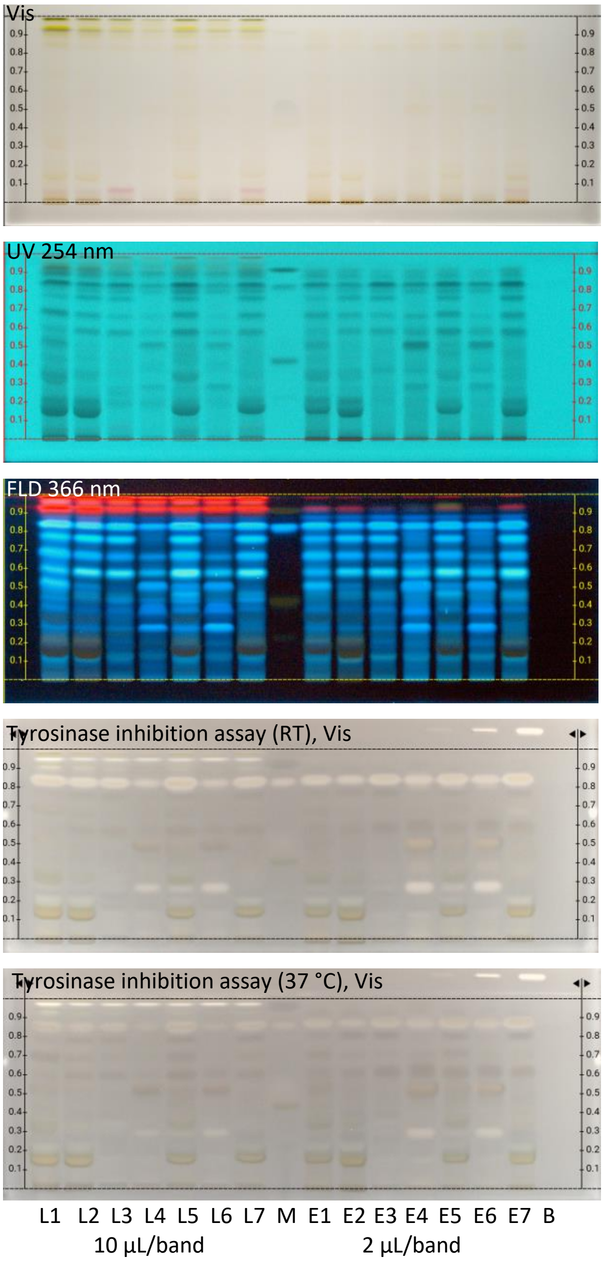

Figure S6 HPTLC–tyrosinase inhibition assay: HPTLC–Vis/UV/FLD chromatograms of 14 peppermint products (Table 1) extracted with ethanol – ethyl acetate – water 1:1:1 (0.1 mg/mL each; 10 µL/band for **L1**–**L7** and 2 µL/band for **E1**–**E7**) on HPTLC plate silica gel 60 F_{254s} along with standard mixture (**M**; eriocitrin, luteolin-7-*O*-glucoside, rosmarinic acid, and apigenin, 1 µg/band each) and the respective solvent blank (**B**) separated with 7 mL ethyl acetate – toluene – formic acid – water 8:2:1.5:1 and detected at white light illumination, UV 254 nm and FLD 366 nm as well as after the tyrosinase inhibition assay comparing incubation at room temperature *versus* 37 °C, detected at white light illumination. The PC kojic acid was applied at 1, 3 and 6 µL/band (0.1 mg/mL in ethanol) in the upper right plate. After 5 days, the assay plate was derivatized via the natural product A reagent detected at FLD 366 nm and white light illumination.

Applied on the tyrosinase inhibition autogram after 5 d

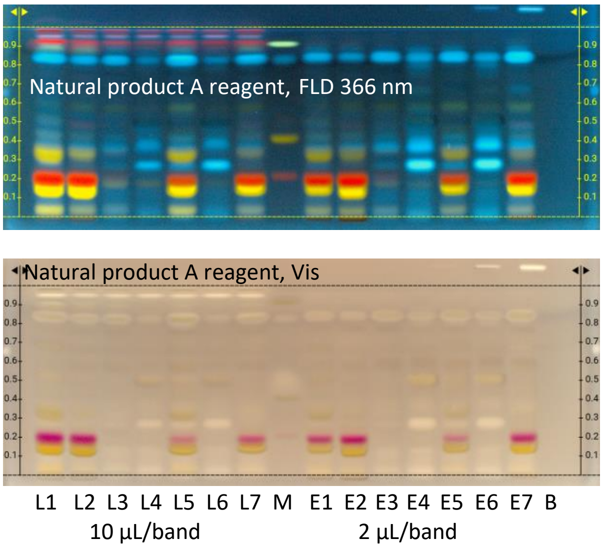

Figure S6 continued

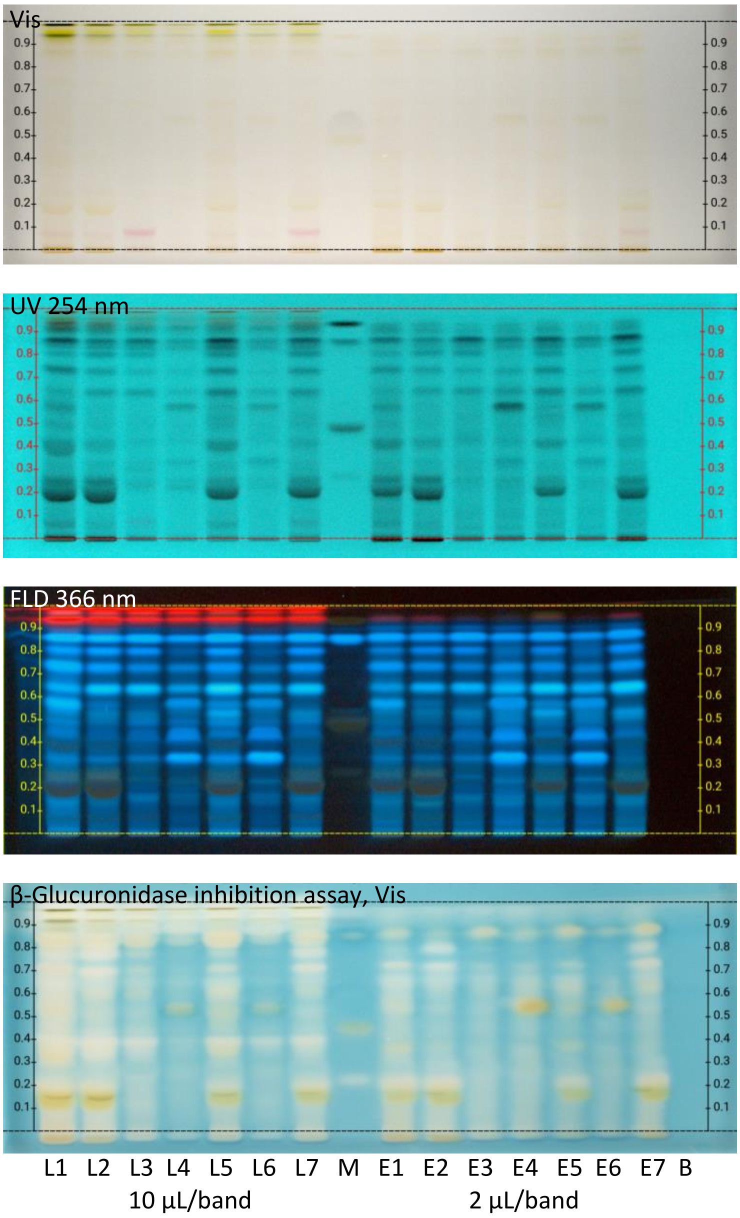

Figure S7 HPTLC–β-glucuronidase inhibition assay: HPTLC–Vis/UV/FLD chromatograms of 14 pepper-mint products (Table 1) extracted with ethanol – ethyl acetate – water 1:1:1 (0.1 mg/mL each; 10 µL/band for **L1**–**L7** and 2 µL/band for **E1**–**E7**) on HPTLC plate silica gel 60 F_{254s} along with standard mixture (**M**; eriocitrin, luteolin-7-*O*-glucoside, rosmarinic acid, and apigenin, 1.5 µg/band each) and the respective solvent blank (**B**) separated with 7 mL ethyl acetate – toluene – formic acid – water 8:2:1.5:1 and detected at white light illumination, UV 254 nm and FLD 366 nm as well as after the β-glucuronidase inhibition assay, detected at white light illumination

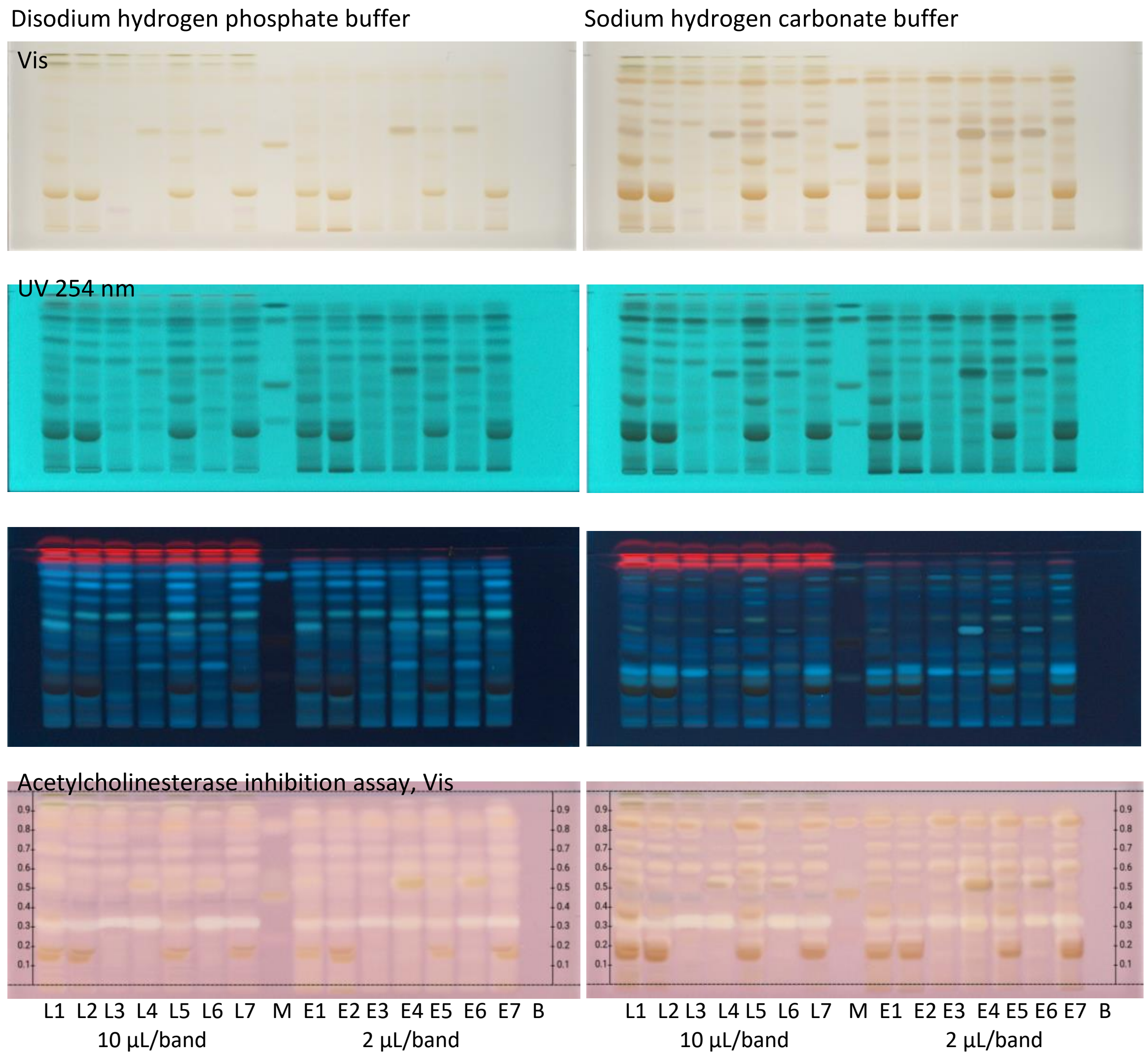

Figure S8 HPTLC–acetylcholinesterase inhibition assay performed using two different buffers, *i.e.* disodium hydrogen phosphate buffer (8 g in 60 mL water, citric acid 0.1 M added to reach pH 7.5, filled up to 100 mL) *versus* sodium hydrogen carbonate buffer (2.5 g in 100 mL water): HPTLC–Vis/UV/FLD chromatograms of 14 peppermint products (Table 1) extracted with ethanol – ethyl acetate – water 1:1:1 (0.1 mg/mL each; 10 µL/band for **L1**–**L7** and 2 µL/band for **E1**–**E7**) on HPTLC plate silica gel 60 F_{254s} along with standard mixture (**M**; eriocitrin, luteolin-7-*O*-glucoside, rosmarinic acid, and apigenin, 1.5 µg/band each) and the respective solvent blank (**B**) separated with 7 mL ethyl acetate – toluene – formic acid – water 8:2:1.5:1 and detected at white light illumination, UV 254 nm and FLD 366 nm as well as after the acetylcholinesterase inhibition assay, detected at white light illumination. The PC rivastigmine was applied at 2, 4 and 8 µL/band (0.1 mg/mL in methanol) in the upper right plate.

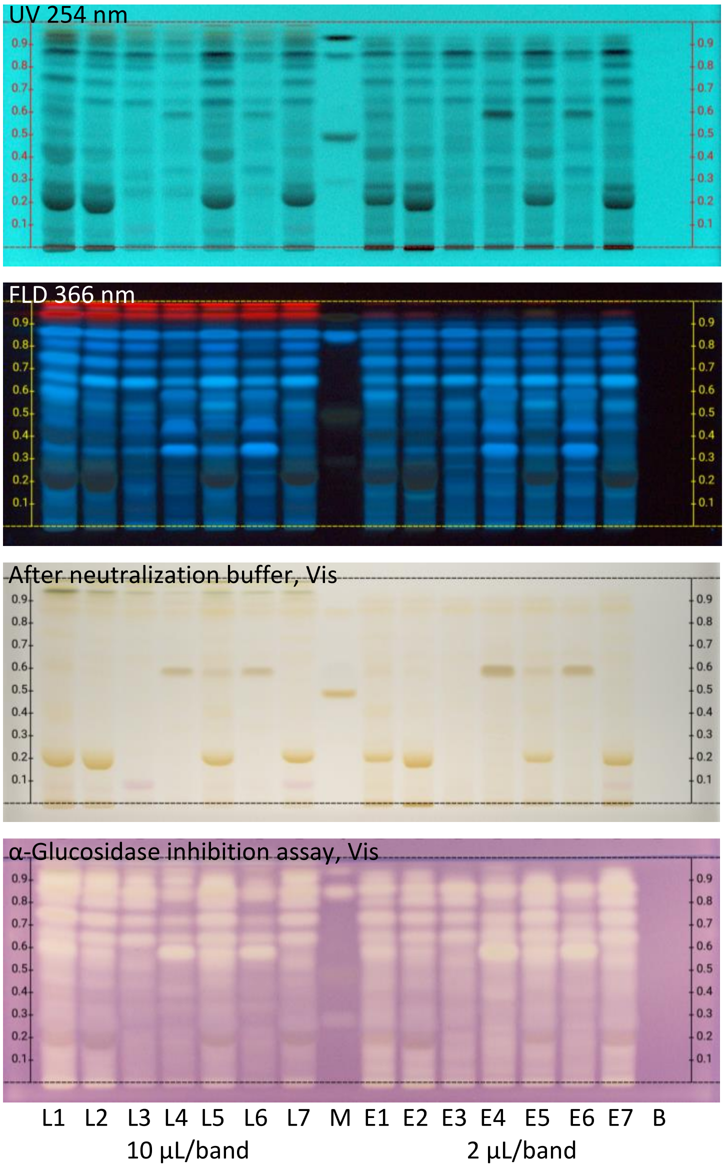

Figure S9 HPTLC–α-glucosidase inhibition assay: HPTLC–Vis/UV/FLD chromatograms of 14 peppermint products (Table 1) extracted with ethanol – ethyl acetate – water 1:1:1 (0.1 mg/mL each; 10 µL/band for **L1**–**L7** and 2 µL/band for **E1**–**E7**) on HPTLC plate silica gel 60 F_{254s} along with standard mixture (**M**; eriocitrin, luteolin-7-*O*-glucoside, rosmarinic acid, and apigenin, 1.5 µg/band each) and the respective solvent blank (**B**) separated with 7 mL ethyl acetate – toluene – formic acid – water 8:2:1.5:1 and detected at white light illumination, UV 254 nm and FLD 366 nm as well as after the α-glucosidase inhibition assay, detected at white light illumination.

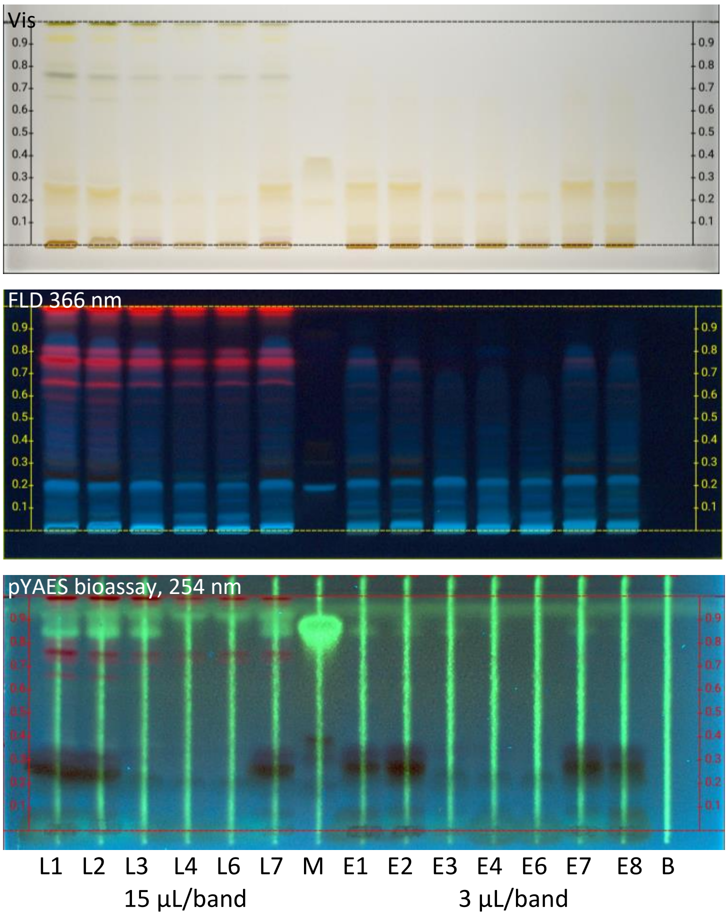

Figure S10 Planar bioprofiling for estrogenic and anti-estrogenic compounds: HPTLC–Vis/UV/FLD chromatograms of peppermint products (Table 1) extracted with ethanol – ethyl acetate – water 1:1:1 (0.1 mg/mL each; 15 µL/band or 1.5 mg for **L1**–**L7**, L5 skipped, and 3 µL/band or 0.3 mg for **E1**–**E8**; E8 instead of E5) on HPTLC plate silica gel 60 along with standard mixture (**M**; eriocitrin, luteolin-7-*O*-glucoside, rosmarinic acid, and apigenin, 2.3 µg/band each) and the respective solvent blank (**B**) separated with 7 mL ethyl acetate – toluene – methanol – water 4:1:1:0.4 and detected at white light illumination and FLD 366 nm as well as after the multiplex pYAES bioassay at 254 nm. There were revealed as green fluorescent band up to two apolar estrogens in the sample and one estrogen (rosmarinic acid) in the standard mixture. Some anti-estrogenic effects were observed as signal reduction of the green fluorescent 17β-estradiol stripe. The green fluorescence of the 17β-estradiol stripe was considered as positive control.

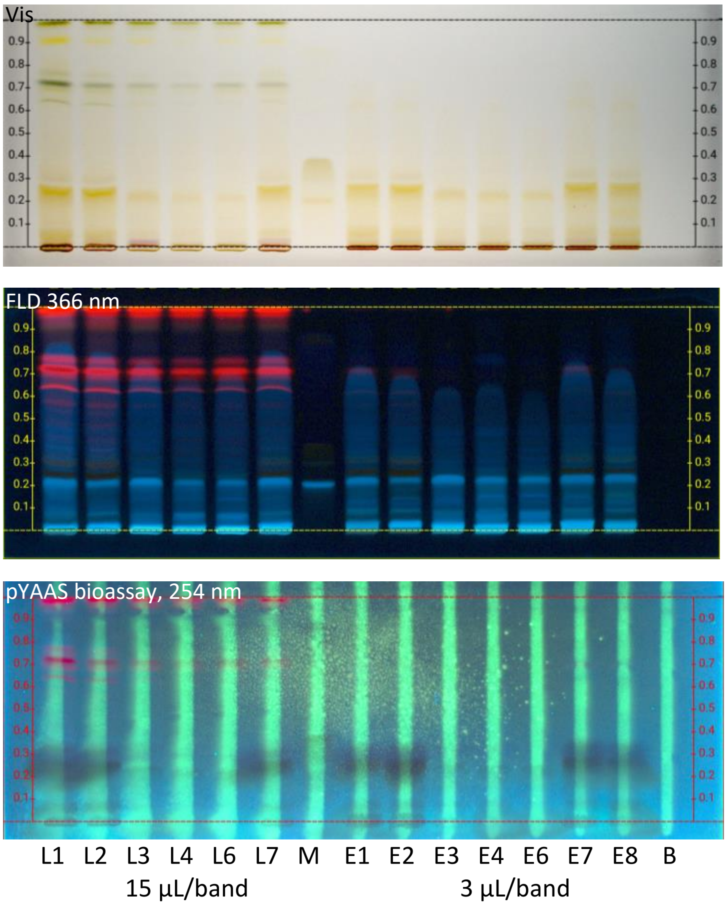

Figure S11 Planar bioprofiling for androgenic and antiandrogenic compounds: HPTLC–Vis/UV/FLD chromatograms of peppermint products (Table 1) extracted with ethanol − ethyl acetate − water 1:1:1 (0.1 mg/mL each; 15 µL/band or 1.5 mg for **L1**–**L7**, L5 skipped, and 3 µL/band or 0.3 mg for **E1**–**E8**; E8 instead of E5) on HPTLC plate silica gel 60 along with standard mixture (**M**; eriocitrin, luteolin-7-*O*-glucoside, rosmarinic acid, and apigenin, 2.3 µg/band each) and the respective solvent blank (**B**) separated with 7 mL ethyl acetate – toluene – methanol – water 4:1:1:0.4 and detected at white light illumination and FLD 366 nm as well as after the multiplex pYAAS bioassay at 254 nm. There were no androgens detected as green fluorescent band despite the high sample load but anti-androgenic effects as signal reduction of the green fluorescent testosterone stripe. The green fluorescence of the testosterone stripe was considered as positive control.

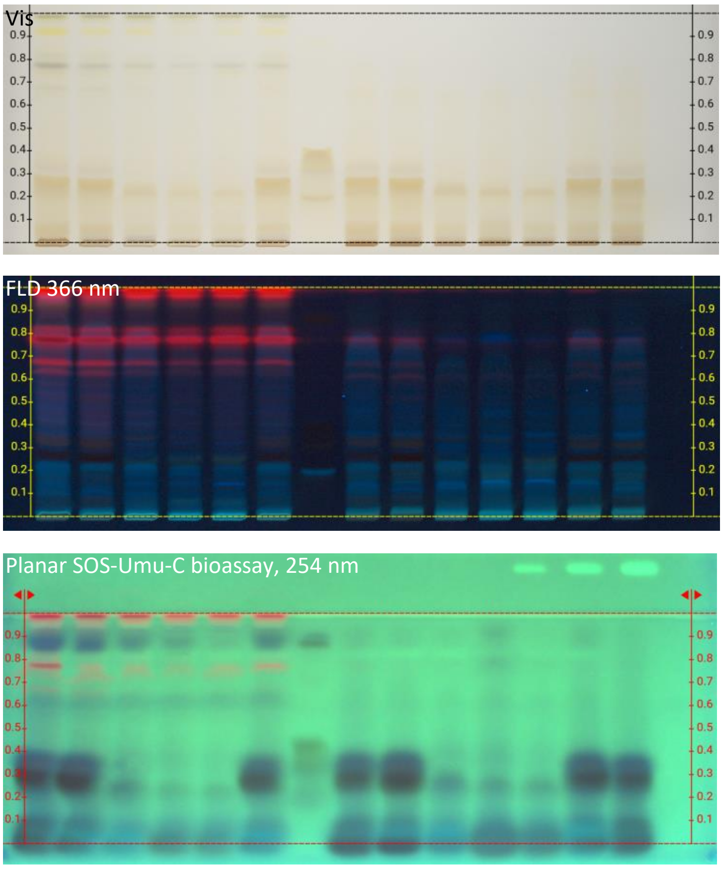

Figure S12 Planar bioprofiling for genotoxic compounds: HPTLC–Vis/UV/FLD chromatograms of peppermint products (Table 1) extracted with ethanol – ethyl acetate – water 1:1:1 (0.1 mg/mL each; 15 µL/band or 1.5 mg for **L1**–**L7**, L5 skipped, and 3 µL/band or 0.3 mg for **E1**–**E8**; E8 instead of E5) on HPTLC plate silica gel 60 along with standard mixture (**M**; eriocitrin, luteolin-7-*O*-glucoside, rosmarinic acid, and apigenin, 2.3 µg/band each) and the respective solvent blank (**B**) separated with 7 mL ethyl acetate – toluene – methanol – water 4:1:1:0.4 and detected at white light illumination and FLD 366 nm as well as after the planar SOS-Umu-C bioassay at 254 nm. The PC 4-nitroquinoline 1-oxide was applied at 0.4, 1 and 2 µL/band (500 pg/µL in methanol) in the upper right plate. There was no green fluorescent genotoxin band revealed in the samples despite the high sample load.

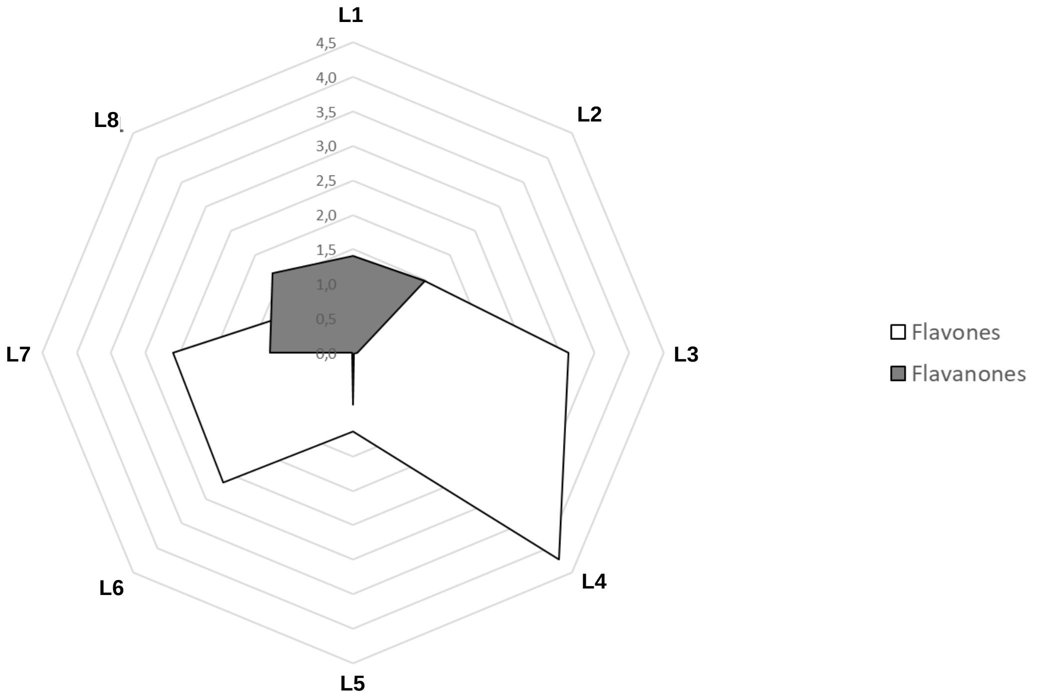

Figure S13. HPLC–PDA spider-web diagram of total content (%, dry basis) of flavones (luteolin-7-*O*-glucuronide, luteolin-7-*O*-rutinoside and isorhoifolin) and flavanones (eriocitrin and eriodictyol-7-*O*-glucoside) of different peppermint leaf samples **L1**–**L8** from USA and Europe (Table 1)

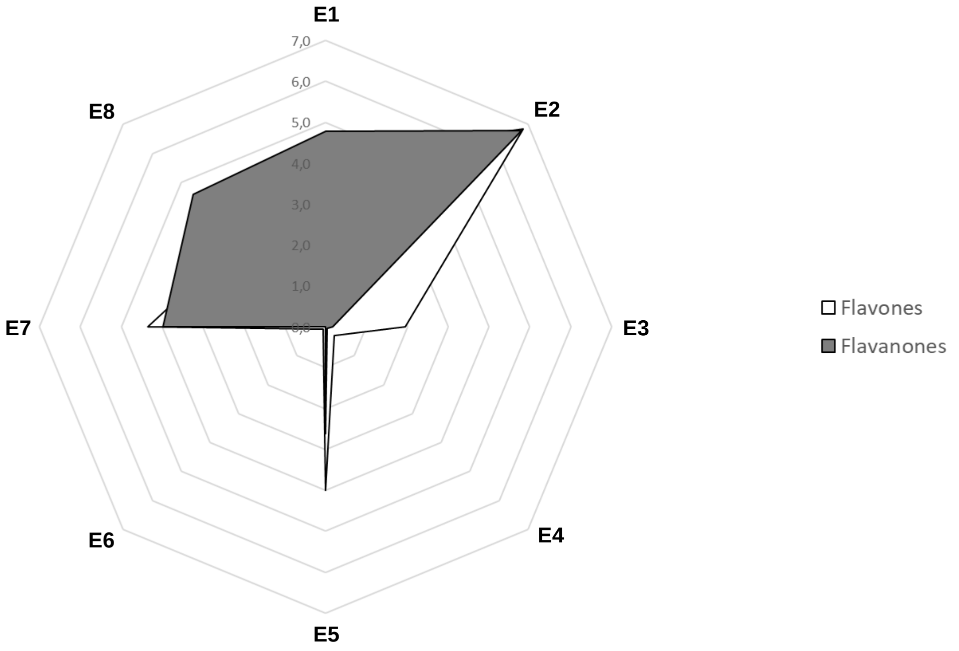

Figure S14. HPLC–PDA spider-web diagram of total content (%, dry basis) of flavones (luteolin-7-*O*-glucuronide, luteolin-7-*O*-rutinoside and isorhoifolin) and flavanones (eriocitrin and eriodictyol-7-*O*-glucoside) of different peppermint leaf samples **E1**–**E8** from USA and Europe (Table 1)

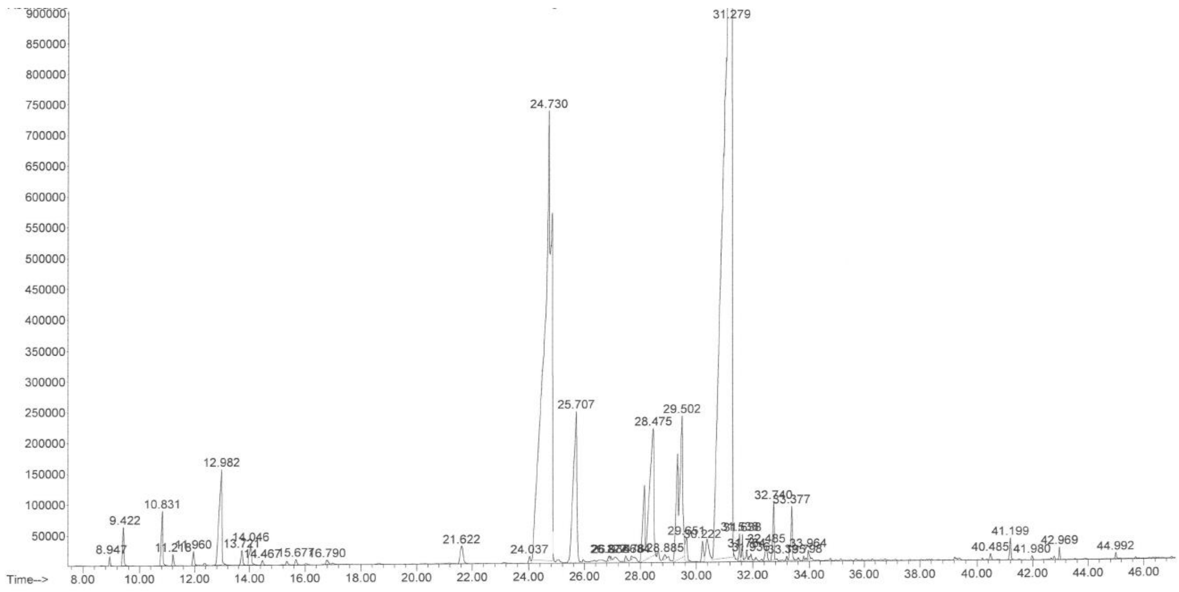

Figure S15. Headspace SPME−GC−FID profile of the proprietary peppermint leaf sample **L5** from USA, showing terpinolene (11.22 min, 15.68 min), limonene (11.96 min), eucalyptol (12.98 min), terpinene (14.05 min), menthone (24.73 min, 25.71 min), menthyl acetate (26.88 min, 28.48 min), benzaldehyde (26.94 min), linalool (27.68 min), β-caryophyllene (28.89 min), neomenthol (29.50 min), menthol (31.28 min, 31.64 min), α-ternineol (32.485 min), and p-menth-1-en-3-one (33.38 min)

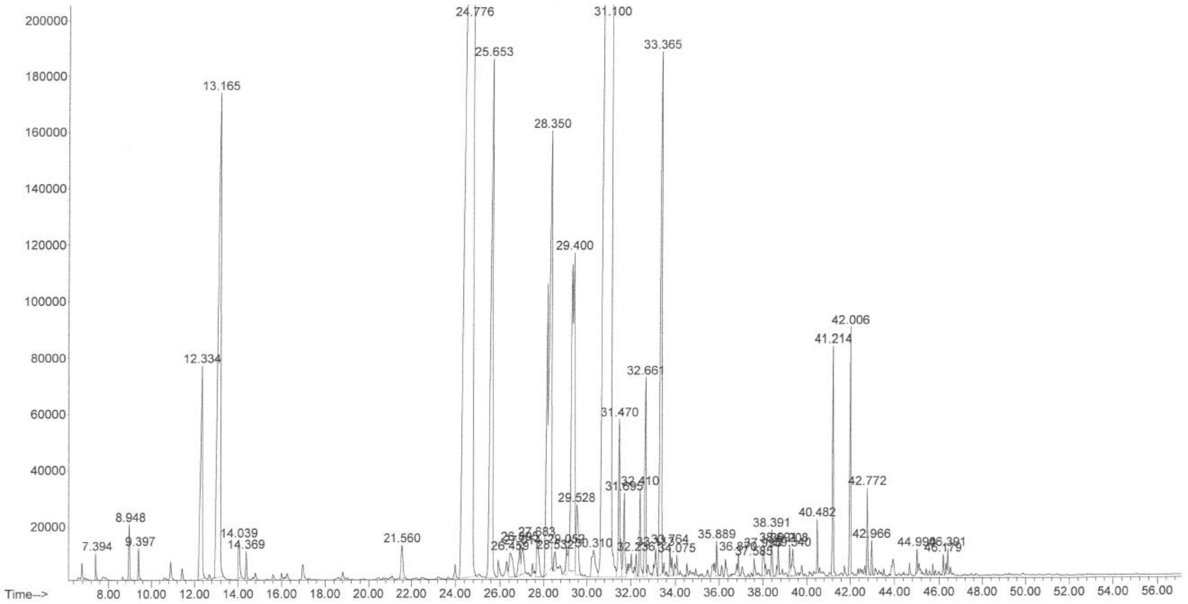

Figure S16. Headspace SPME−GC−FID profile of the European peppermint leaf sample **L2**, showing limonene (12.33 min), eucalyptol (13.17 min), terpinene (14.37 min), menthone (24.77 min, 25.65 min), menthyl acetate (26.89 min, 28.35 min), isomenthyl acetate (27.01 min), linalool (27.68 min), isopulegol (28.53 min), β-caryophyllene (29.05 min), neomenthol (30.31), menthol (31.10 min, 31.64 min), α-ternineol (32.41 min), p-menth-1-en-3-one (33.37 min), piperitone (37.99 min), and eugenol (42.77 min)

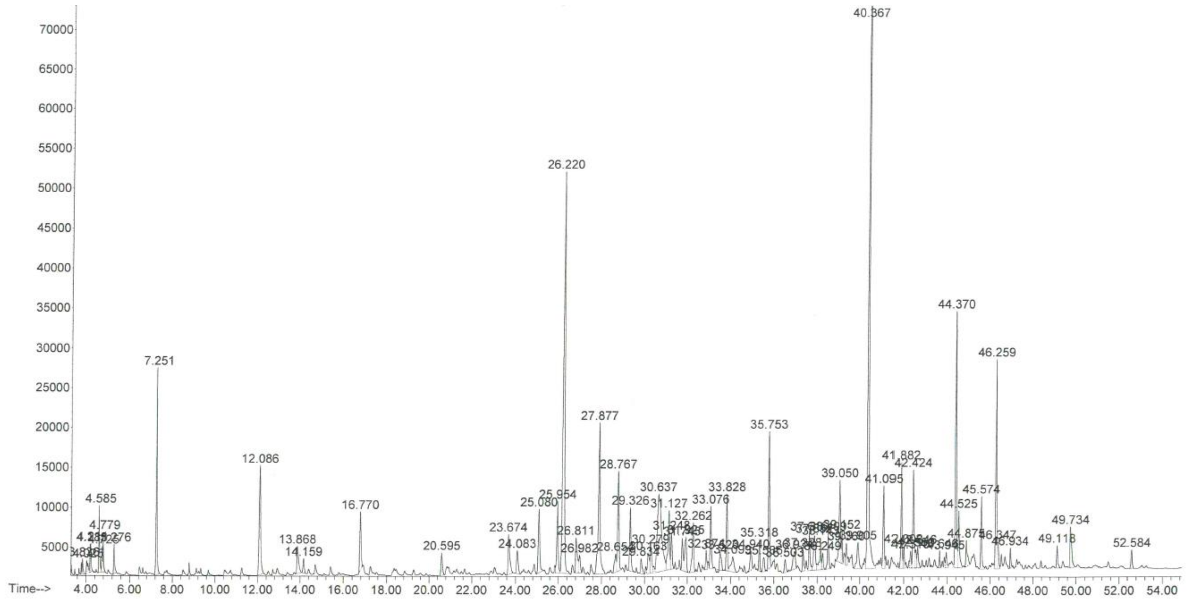

Figure S17. Headspace SPME–GC–FID profile of the European peppermint extract sample **E2**, showing limonene (12.09 min), hex-2-enal (13.87 min), terpinene (14.16 min), menthone (24.08 min), benzaldehyde (26.81 min), *p*-menth-4-en-3-one (26.98 min), menthyl acetate (27.88 min, 28.48 min), β-caryophyllene (28.77 min), *p*-mentha-2,8-dien-1-ol (30.16 min), menthol (30.64 min), and *p*-menth-1-en-3-one (33.07 min)

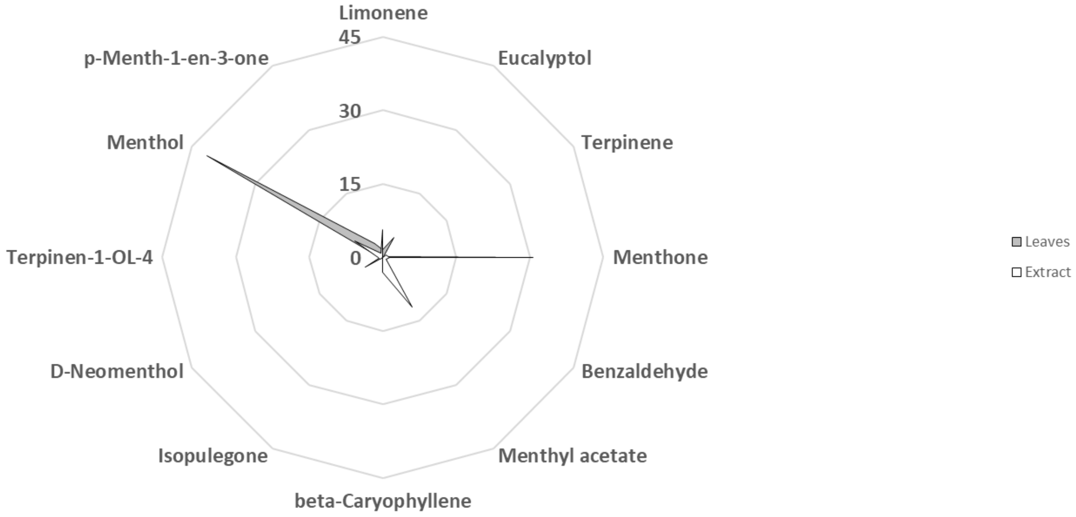

Figure S18 Headspace SPME–GC–FID relative response proportion (%) of major volatile components found in the European peppermint leaf sample **L2** and the corresponding extract **E2**

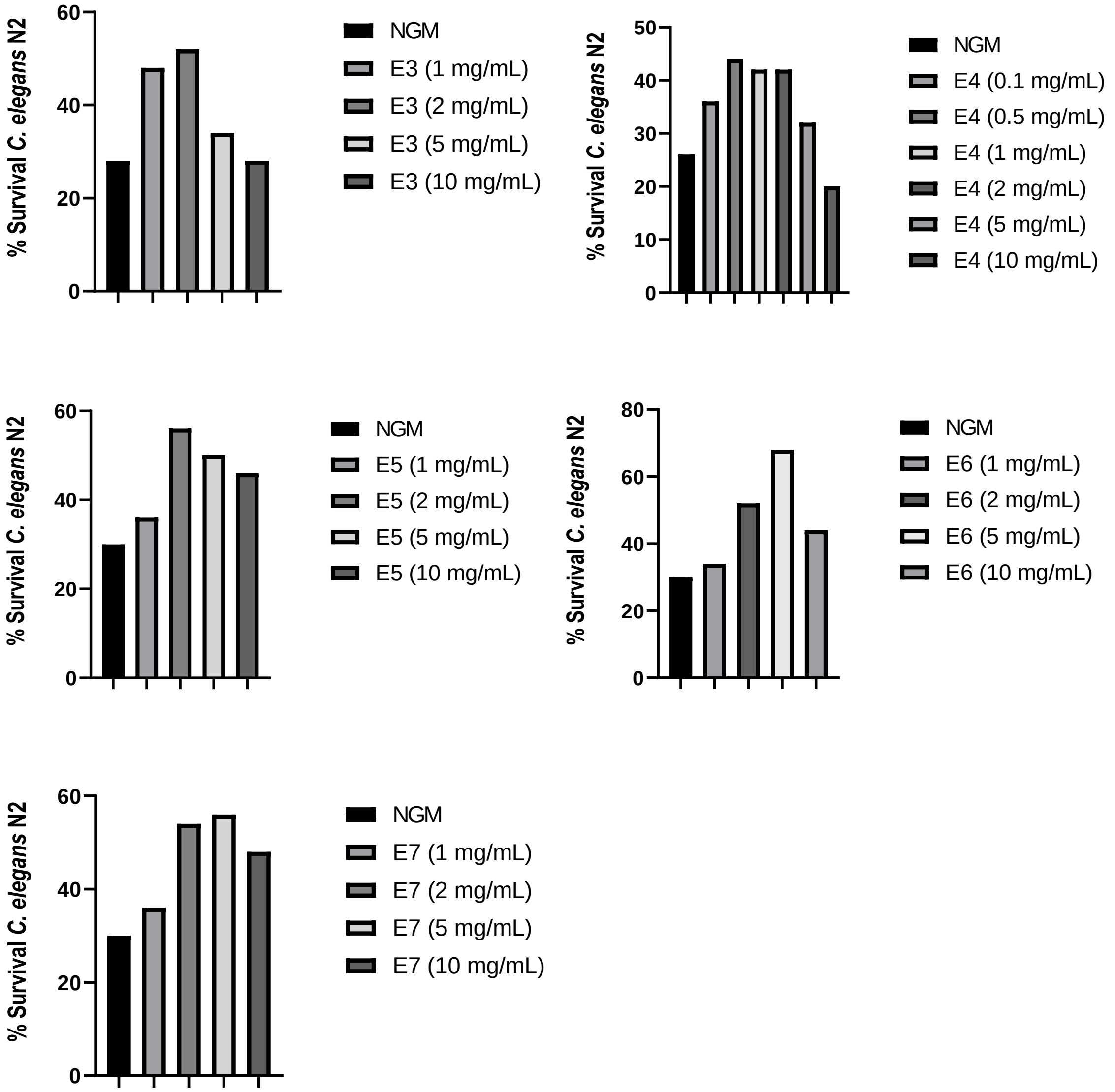

Figure S19 Dose-response of the peppermint extract samples **E1**–**E7** on the antioxidant activity in the *C. elegans* model. Worms were fed with peppermint extract samples at different doses, and after exposure to an acute oxidative stress, the percentage of worm's survival was determined.

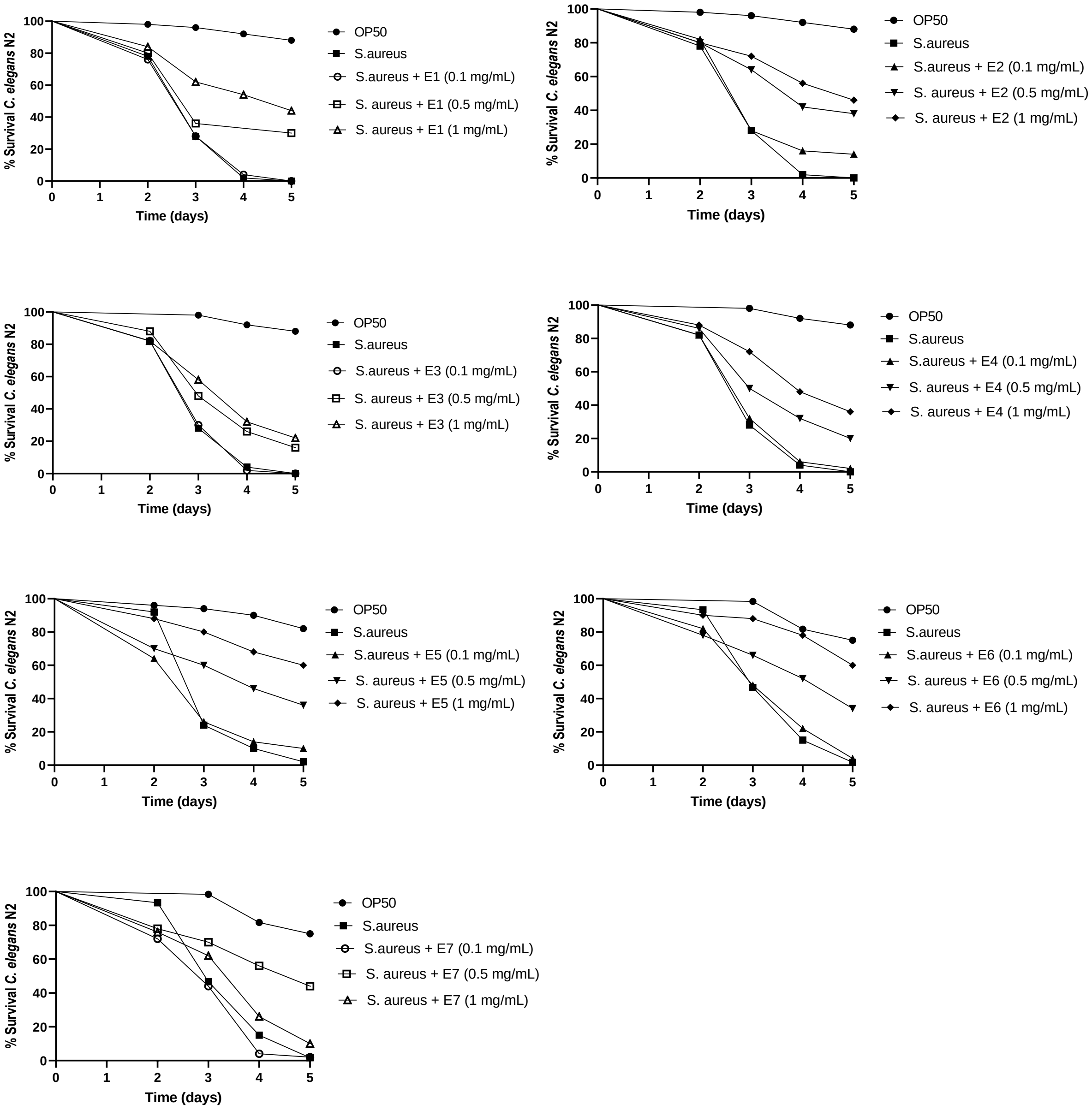

Figure S20 Dose-response of the peppermint extract samples**E1–E7** on the pathogen protection in the *C. elegans* model. Worms were fed with peppermint extract samples at different doses and infected with a lawn of *S. aureus* ATCC 25923. The percentage of alive worms determined every day is represented.

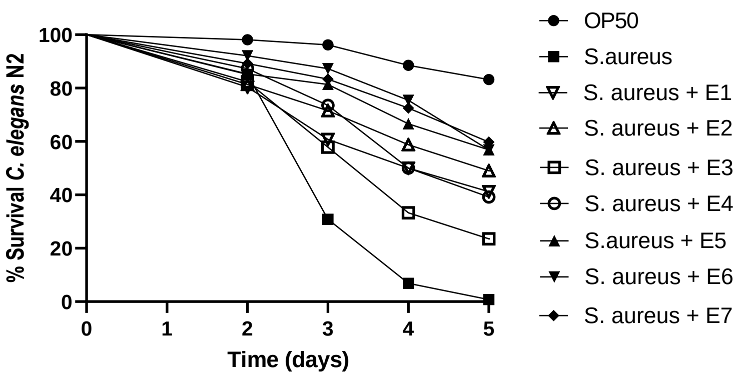

Figure S21. Percentage of survival of *C. elegans* N2 treated with the peppermint extract samples **E1–E7** and infected with the pathogen *S. aureus* (ATCC 25923). p-Values < 0.0001 for all samples compared to control-infected condition. Log Rank T-test was applied. Data are the average of two independent assays.

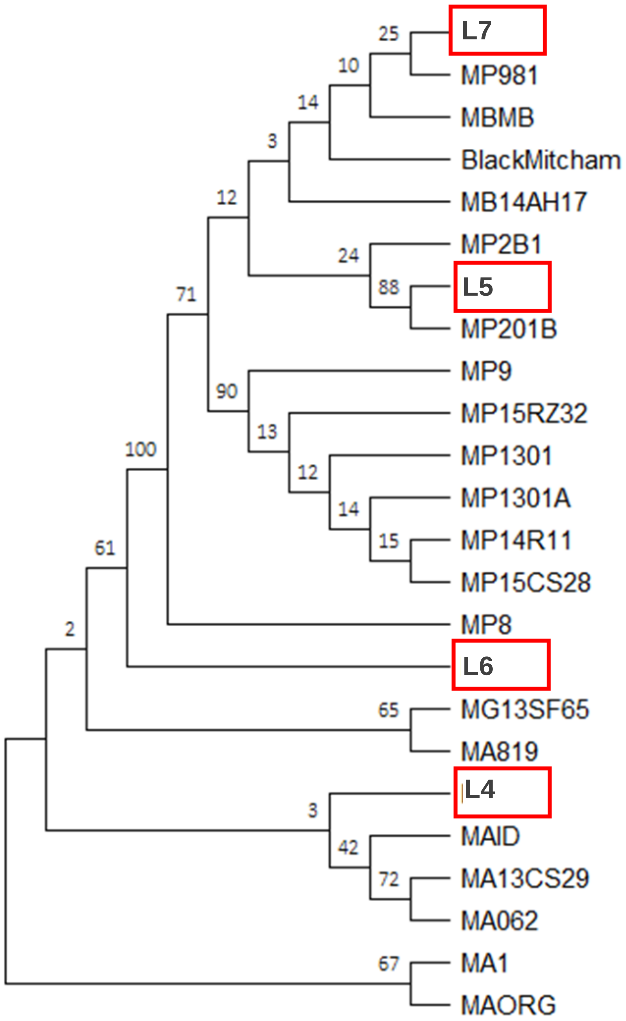

Figure S22. Phylogenetic relationships of the 24 mint varieties inferred using the maximum parsimony. The percentage of replicate trees in which the associated varieties clustered together in the bootstrap test (500 replicates) were shown next to the branches.
